# The medaka novel immune-type receptor (NITR) gene clusters reveal an extraordinary degree of divergence in variable domains

**DOI:** 10.1186/1471-2148-8-177

**Published:** 2008-06-19

**Authors:** Salil Desai, Amy K Heffelfinger, Timothy M Orcutt, Gary W Litman, Jeffrey A Yoder

**Affiliations:** 1 Department of Molecular Biomedical Sciences and Center for Comparative Medicine and Translational Research, College of Veterinary Medicine, North Carolina State University, 4700 Hillsborough Street, Raleigh, NC 27606, USA; 2 Immunology Program, North Carolina State University, 4700 Hillsborough Street, Raleigh, NC 27606, USA; 3 Department of Pediatrics, University of South Florida College of Medicine, 830 First Street South, St. Petersburg, FL 33701, USA; 4 H. Lee Moffitt Cancer Center and Research Institute, 12902 Magnolia Avenue, Tampa, FL 33612, USA; 5 Department of Molecular Genetics, All Children's Hospital, 801 Sixth Street South, St. Petersburg, FL 33701, USA

## Abstract

**Background:**

Novel immune-type receptor (NITR) genes are members of diversified multigene families that are found in bony fish and encode type I transmembrane proteins containing one or two extracellular immunoglobulin (Ig) domains. The majority of NITRs can be classified as inhibitory receptors that possess cytoplasmic immunoreceptor tyrosine-based inhibition motifs (ITIMs). A much smaller number of NITRs can be classified as activating receptors by the lack of cytoplasmic ITIMs and presence of a positively charged residue within their transmembrane domain, which permits partnering with an activating adaptor protein.

**Results:**

Forty-four NITR genes in medaka (*Oryzias latipes*) are located in three gene clusters on chromosomes 10, 18 and 21 and can be organized into 24 families including inhibitory and activating forms. The particularly large dataset acquired in medaka makes direct comparison possible to another complete dataset acquired in zebrafish in which NITRs are localized in two clusters on different chromosomes. The two largest medaka NITR gene clusters share conserved synteny with the two zebrafish NITR gene clusters. Shared synteny between NITRs and CD8A/CD8B is limited but consistent with a potential common ancestry.

**Conclusion:**

Comprehensive phylogenetic analyses between the complete datasets of NITRs from medaka and zebrafish indicate multiple species-specific expansions of different families of NITRs. The patterns of sequence variation among gene family members are consistent with recent birth-and-death events. Similar effects have been observed with mammalian immunoglobulin (Ig), T cell antigen receptor (TCR) and killer cell immunoglobulin-like receptor (KIR) genes. NITRs likely diverged along an independent pathway from that of the somatically rearranging antigen binding receptors but have undergone parallel evolution of V family diversity.

## Background

The two major forms of novel immune-type receptors (NITRs) possess either one N-terminal ectodomain of the Ig variable (V) type, which may contain a joining (J) or J-like sequence or an N-terminal V domain plus a second C-terminal Ig domain of the intermediate (I) type. Most NITRs are predicted to encode type I transmembrane cell surface proteins that possess extracellular immunoglobulin (Ig) domains and can be classified as inhibitory or activating. A smaller number of NITR genes lack a transmembrane domain and are predicted to be secreted proteins [1,2]. NITRs are expressed in several different hematopoietic lineages [3,4] and their overall structure and cytoplasmic signaling resemble the mammalian natural killer cell receptors (e.g. KIRs) [1,5,6]. NITRs have been reported to bind allogeneic cell surface targets, although their precise ligand specificity is not clear [Cannon et al., unpublished observations and [7]]. Other observations, including upregulation of transcription in response to pathogens, support a role for NITRs in immunity [8].

NITRs were identified initially in the compact genome of the Southern pufferfish (*Spheroides nephelus*); more than 26 NITR genes were shown to be encoded in a single gene cluster [9,10]. Subsequent analysis of the zebrafish (*Danio rerio*) genome revealed one large NITR gene cluster encoding 36 NITRs on chromosome 7 and a second smaller NITR gene cluster encoding 3 NITRs on chromosome 14 [2,11]. NITR cDNAs also have been characterized from channel catfish (*Ictalurus punctatus*), rainbow trout (*Oncorhynchus mykiss*) and Japanese flounder (*Paralichthys olivaceus*) [3,4,12,13] and it is assumed that they are present in all bony fish. The majority of NITRs possess cytoplasmic immunoreceptor tyrosine-based inhibition motifs (ITIMs); inhibitory functions have been confirmed *in vitro *[14]. A smaller number of NITRs possess a positively charged residue within their transmembrane domain and have been shown to interact with Dap12 through which they mediate an activating function [15].

We herein report the identification of 44 medaka (*Oryzias latipes*) NITR genes, which can be classified into 24 families including inhibitory and activating forms; this represents nearly twice the number of NITR families that have been identified in any other species. The resolution of this dataset of NITRs along with the previous genomic resolution of NITRs in zebrafish permits a comprehensive phylogenetic analysis as well as the examination of shared synteny. The NITRs emerge as independent families of immune-type receptors in which the evolution of the V regions exhibit considerable similarity to that seen in multigene families encoding Ig, TCRs and KIRs.

## Results

### Medaka encode 44 NITRs on three chromosomes

**Figure 1:**
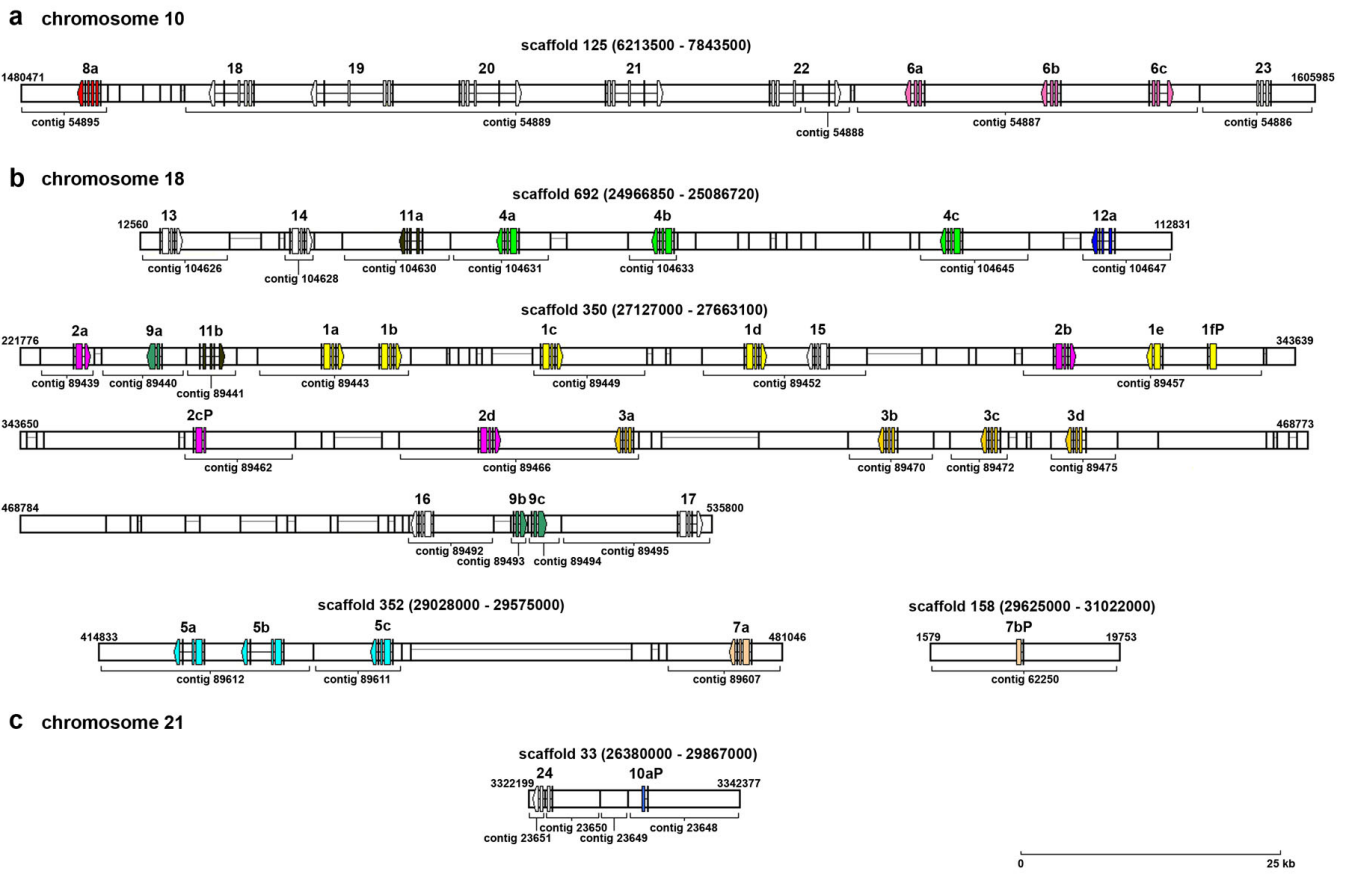
**Genomic organization of medaka NITRs**. Predicted exon organization of medaka NITRs mapped to chromosome: **(a) **10, **(b) **18 and **(c) **21; genome scaffold numbers (above), contig numbers (below). Gene numbers are indicated (e.g. *NITR8a *is shown as "8a"). Exons are represented by rectangles; arrowhead indicates transcriptional orientation. Color-coding designates gene family and corresponds to Figure 4; singletons are white. Horizontal gray lines represent gaps between contigs. *NITR8b*, *NITR10b*, and *NITR12b *are encoded by contigs 121976, 121209 and 118885, respectively, and are not yet placed in the genome.

The reduced levels of protein sequence identity between NITRs found in different species make it difficult to identify genetic orthologs between fish species outside of conserved core residues. In order to identify NITR genes from medaka, we data mined the recently released medaka genome database [16,17]. By using zebrafish NITR sequences as queries, it was possible to identify a number of candidate genes that encode Ig domains. By then restricting the queries only to genes that encode an authentic NITR I-type domain [14], NITR gene clusters were identified on medaka chromosomes 10, 18 and 21. The search was then directed to include all genes predicted to encode V domains in these genomic regions. In total, 10 NITR genes were identified on chromosome 10; 30 NITR genes and three pseudogenes (defined as possessing a stop codon within the V or I domain) were identified on chromosome 18; and one NITR gene and one pseudogene were identified on chromosome 21 (Figure 1 and Additional File 1). Three additional NITR genes (*NITR8b*, *NITR10b *and *NITR12b*) cannot be assigned to a specific chromosome. It is possible that certain genes described here may represent allelic variants and that additional NITR genes may be identified in future releases of the medaka genome.

**Figure 2:**
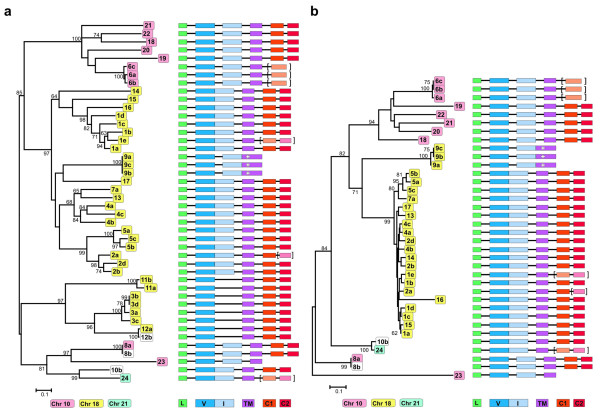
**Phylogenetic relationships between medaka NITR V and I domains**. **(a – left panel) **Neighbor-joining tree of V domains encoded by 44 medaka NITR genes. NITR numbering corresponds to Figure 1. Bootstrap values are assigned to each interior branch. Bootstrap values less than 60 are not shown. Branch lengths correspond to the number of amino acid substitutions estimated by Poisson correction; scale indicated below. Color-coding (below) corresponds to chromosomal placement; white is used if locus is not assigned. **(right panel) **Generalized genomic organization of NITRs shown in left panel (summarized from Figure 1, not to scale). Rectangles correspond to exons (L = leader; V = variable Ig domain; I = intermediate Ig domain; TM = transmembrane domain; C1 and C2 = cytoplasmic domain). Predicted but unidentifiable exons are indicated in parentheses. **(b – left panel) **A neighbor-joining tree of I domains encoded by 36 medaka NITR genes. **(right panel) **Generalized genomic organization of NITRs shown in left panel (summarized from Figure 1, not to scale). Annotation as in (a).

The 44 NITR genes that were identified can be grouped into 24 V families, based on >70% predicted amino acid sequence identity (Figure 2a and Additional File 2). Twelve families consist of multiple (up to five) members and the remaining families are singletons. Each NITR family was assigned a number and for families with multiple members, each member was assigned a letter (e.g. NITR1a, NITR1b, etc.) as described [2]. With the exception of the NITR9 family (see below) in which activating potential (rather than V structure) served as the basis for assignment to a specific gene family, unequivocal orthologs between the NITRs of medaka and any other species were not identified and the numbering of these genes was arbitrary.

### Two major classes of NITR gene organization

All of the NITR genes currently described from the Southern pufferfish and 11 NITR genes from a second pufferfish species, *Tetraodon nigroviridis *(Additional file 3), encode two Ig domains (V and I domains) in a single exon [10]. In contrast, all of the two-Ig domain NITR genes described from zebrafish encode the V and I domains in two exons [2]. Both types of NITR gene organization are present in medaka (Figure 2). The medaka NITR genes encoding two Ig domains in a single exon are all located on chromosomes 18 and 21; the medaka NITR genes encoding two Ig domains in two exons are located on chromosome 10 with the exception of the *NITR9 *genes, which are on chromosome 18.

Twenty-one full-length NITR cDNAs were cloned from medaka kidney and spleen by 3' RACE using nested primers that include the predicted translational start codon (Additional Files 4 and 5) in order to verify the exon-intron boundaries for the NITRs. The predicted exon-intron boundaries most often were verified; however, in several cases, predicted mRNA splice sites were inexact, reflected slight differences in cDNA sequence. It is likely that the genome from inbred Hd-rR medaka and cDNAs from the out-bred orange-red medaka line possess different alleles for certain NITRs. For example, the identification of a *NITR6 *cDNA from the orange-red line confirms that the *NITR6 *genes possess a cytoplasmic tail; however, the cDNA cytoplasmic sequence is too divergent to confidently predict these exons from the Hd-rR genome. In addition the orange-red *NITR12 *cDNA encodes additional nucleotide sequences that are not present in the Hd-rR genome resulting in a stop codon preventing translation of an ITIM-like sequence (itim) in the cDNA (Additional File 6).

### Structural features of medaka NITRs

**Figure 3:**
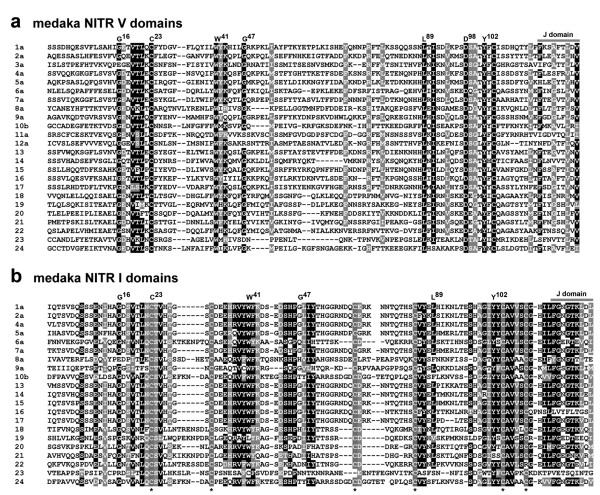
**Conserved features of Medaka NITRs**. Peptide sequence alignment of medaka NITR **(a) **V and **(b) **I regions. Sequences represent the individual NITR families. Identical residues highlighted in black; functionally similar residues highlighted in grey. J-related domain and highly conserved Ig domain residues [1] are indicated; IMGT numbering system is applied [32]. Highly conserved cysteines, representative of NITR I domains, are indicated (*) below the alignment [1].

**Figure 4:**
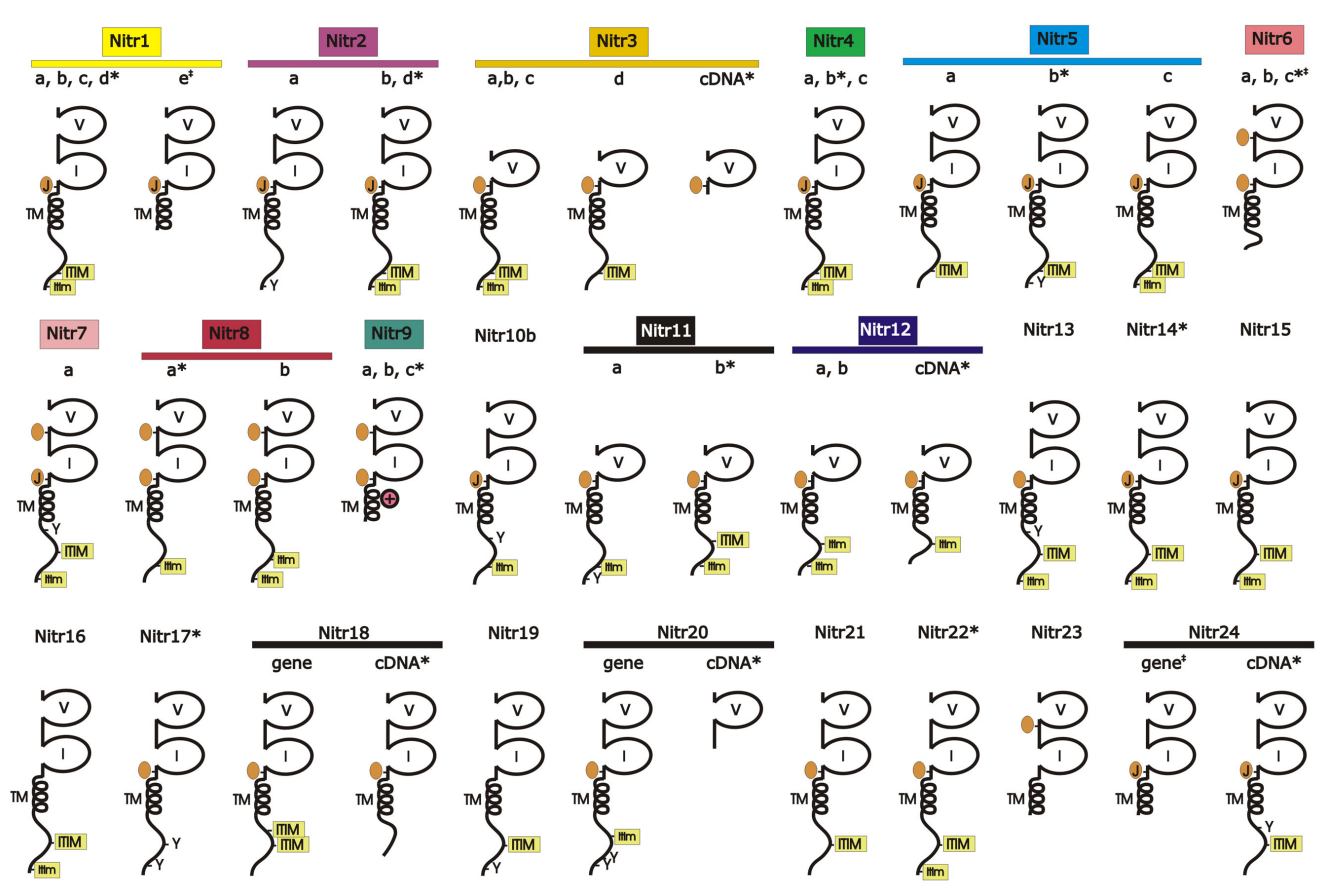
**Predicted protein structures of medaka NITRs**. Schematic representation of 24 NITR families in medaka. V = variable Ig domain; I = intermediate Ig domain; TM = transmembrane region; ⊕ = positively charged residue mid-membrane; orange oval-J = prototypic joining-like region; orange oval = J-like variant (see Additional File 7); ITIM = conventional ITIM and itim = conventional ITIM variant (see Additional File 8). (*) = corresponding full-length cDNA; (I) = cytoplasmic exons unidentifiable; other structures are predicted from genomic sequence. Color-coding as in Figure 1.

The initial search criteria for identifying medaka NITRs was directed to the I domain, which possesses six highly conserved cysteines. NITR22 and NITR23 lack the sixth and third cysteines, respectively (Figure 3). A joining (J; FGXGTXLXV) or J-like peptide sequence at the carboxyl-terminus of either the V and/or the I domain is a conserved feature of nearly all medaka NITRs (Figures 3 and 4 and Additional File 7). The majority of NITRs in medaka and other species can be classified as inhibitory based on the presence of one or two cytoplasmic ITIM or itim sequences (Figure 4 and Additional File 8) as well as by the absence of a charged transmembrane residue, a conserved feature of activating receptors. In medaka, three closely related NITR genes (*NITR9a*, *NITR9b *and *NITR9c*) possess a charged residue (Arg) within their transmembrane domain corresponding to activating NITRs in other species such as *Nitr9*, the single activating NITR gene in zebrafish (Additional File 9) [2,15].

### NITR gene clusters in medaka and zebrafish share conserved synteny

**Figure 5:**
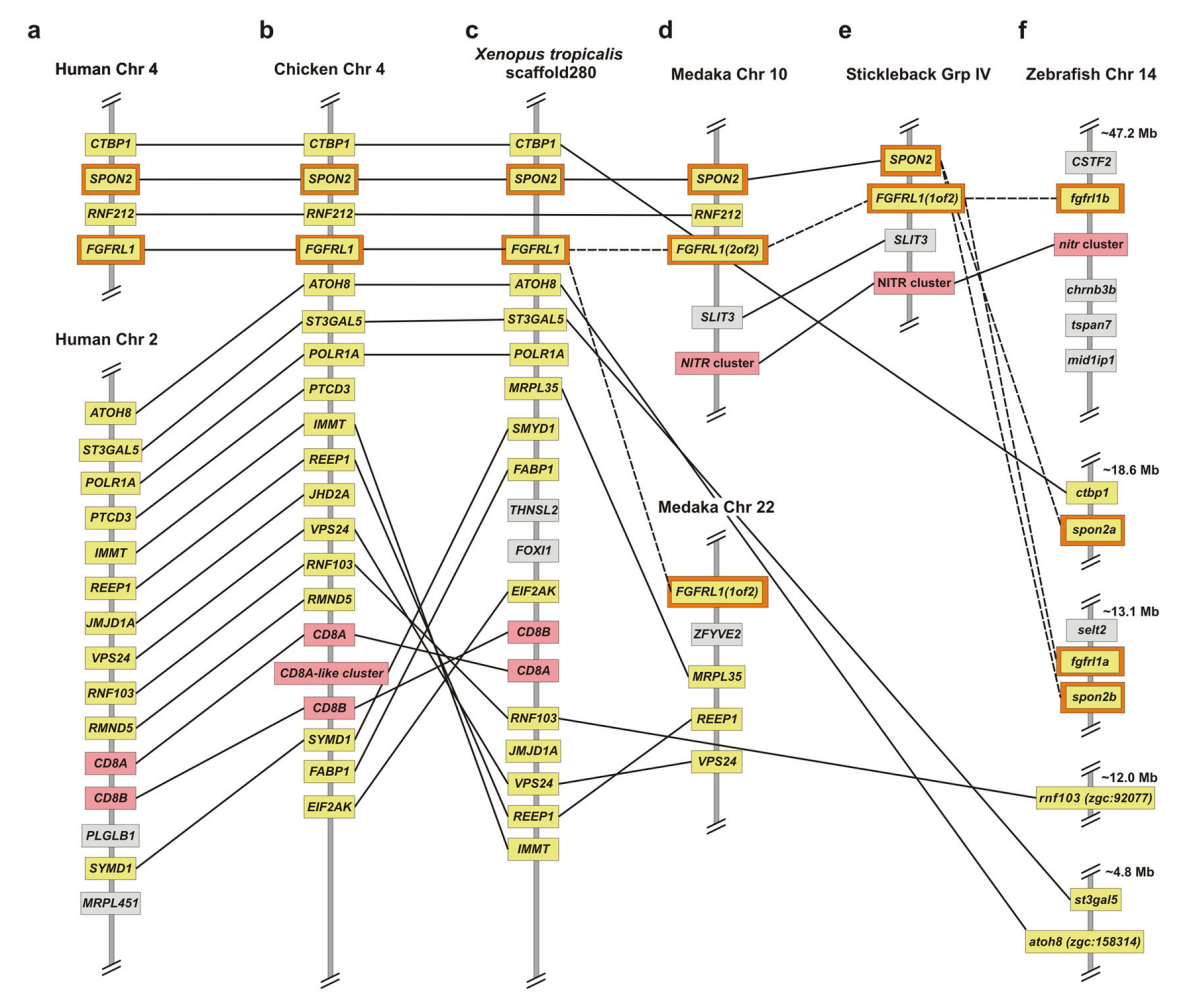
**The *SPON2 and FGFRL1 *genes are linked to *CD8A *in chicken and *Xenopus *and linked to a NITR gene cluster in medaka, zebrafish and stickleback**. **(a) ***SPON2, FGFRL1 *(orange outline) and *CD8A/CD8B *genes (pink background) are not physically linked in human but are linked in **(b) **chicken and **(c) ***Xenopus*. *SPON2 *and *FGFRL1 *orthologs in **(d) **medaka, **(e) **stickleback and **(f) **zebrafish are not linked to *CD8A/CD8B*, but are linked to a NITR gene cluster. *FGFRL1(1of2) *and *FGFRL1(2of2) *in medaka and stickleback and *fgfrl1a *and *fgfrl1b *in zebrafish represent paralogs of FGFRL1. Note the expansion of CD8A-like genes in chicken [22]. Gene names and chromosomal order were identified via Contig View within the Ensembl Genome Browser [28], with the exception of *Xenopus CD8B*. Data were acquired using release NCBI 36 of the human genome, release 2.1 of the chicken genome, release 4.1 of the *Xenopus tropicalis *genome, release 1.0 of the medaka genome and release Zv7 of the zebrafish genome. The location of *Xenopus tropicalis CD8B *was determined by Jacques Robert and Alexander Taranin (personal communication).

The complete annotation of NITRs in zebrafish and medaka permits the investigation of potential shared synteny. The larger zebrafish NITR gene cluster on chromosome 7 is adjacent to the *map4k2l*, *men1 *and *rtn3 *genes [2] and the larger medaka NITR gene cluster is linked to these genes on chromosome 18 (Table 1). The second smaller zebrafish NITR gene cluster on chromosome 14 is adjacent to the *fgfrl1b *gene [11] and the medaka NITR gene cluster on chromosome 10 is tightly linked to *FGFRL1(2of2) *within the same contig (Figure 5). (Medaka and zebrafish possess two *FGFRL1 *orthologs referred to as *fgfrl1a *and *fgfrl1b *in zebrafish and *fgfrl1(1of2) *and *fgfrl1(2of2) *in medaka). Medaka orthologs of additional genes adjacent to the NITR gene cluster on zebrafish chromosome 14 are also encoded on medaka chromosome 10 (Table 1). These observations indicate that the NITR gene clusters on medaka chromosome 18 and zebrafish chromosome 7 share conserved synteny whereas the NITR gene cluster on medaka chromosome 10 and zebrafish chromosome 14 share conserved synteny. The origin of the third, smaller NITR gene cluster on medaka chromosome 21 is not evident, as zebrafish only possess two NITR gene clusters.

**Table 1:** Evidence for conserved synteny between NITR gene clusters

Zebrafish Chr	Zebrafish gene	Medaka ortholog	Medaka Chr
7	*htatip2*	*HTATIP2*	3
7	*zgc:112498*	*PRMT3*	3
7	*slc6a5*	*SLC6A5*	3
7	**NITR cluster**	**NITR cluster**	18
7	*map4k2l*	*MAP4K2*	18
7	*men1*	*MEN1*	18
7	*rtn3*	*Q6IEI5_ORYLA*	18
			
14	*tspan7*	*TSPAN6*	10
14	*chrnb3b*	*CHRNB3 (2 of 2)*	10
14	*LOC555254*	*ITGA6*	10
14	**NITR cluster**	**NITR cluster**	10
14	*fgfrl1b*	*FGFRL1 (2 of 2)*	10
14	*cstf2*	*CSTF2*	10
14	*xkrx*	*XKR3*	10

Orthologous genes were defined by Ensembl. Zebrafish gene order was determined with version Zv6 of the zebrafish genome and Ensembl.

### A bony fish *SPON2/FGFRL1/NITR *locus shares conserved synteny with the *SPON2/FGFRL1/CD8 *locus in *Xenopus *and chicken

NITRs have not been identified outside of bony fish and their evolutionary origins remain unresolved. The genomes of human, chicken, *Xenopus tropicalis *and a third fish species stickleback (*Gasterosteus aculeatus*) were queried in an effort to identify genomic regions, which share conserved synteny with the NITR gene clusters. In medaka, zebrafish and stickleback, one NITR gene cluster is linked to *SPON2 *and *FGFRL1 *orthologs. In chicken and *Xenopus *the *SPON2 *and *FGFRL1 *genes are physically linked to the *CD8A/CD8B *locus (Figure 5). *SPON2*/*FGFRL1 *and *CD8A/CD8B *genes are on different chromosomes in human. In medaka, zebrafish and stickleback the *CD8A/CD8B *locus does not display conserved synteny to human (Additional File 10). The observation that *SPON2 *and *FGFRL *are linked to the *CD8 *locus in chicken and *Xenopus *and that *SPON2 *and *FGFRL *orthologs are linked to a NITR gene cluster in medaka, zebrafish and stickleback is consistent with a common ancestry for these immune-type molecules.

### Medaka *NITR24 *is tightly linked to a *CTLA4 *ortholog

The *NITR24 *locus on medaka chromosome 21 is separated from the V-domain receptor *CTLA4 *by ~0.1 Mb. A small number of predicted olfactory receptor genes lie between CTLA4 and NITR24 (Additional File 11). Although mammalian CTLA4 is tightly linked to CD28, a structurally similar receptor, these genes do not appear to be closely linked in bony fish [18]. Neither NITR gene cluster in zebrafish is linked to CTLA4 or CD28.

### Species-specific expansions of NITR families

In general, most NITRs from a single species lack clear orthologs in other species. The absence of such comparison sets skews multi-species phylogenetic comparisons as not all NITR members from each species (and family) have been identified. Three major inferences can be made from the data described here (Additional File 12) and other observations predicted previously: 1) the activating receptors, medaka NITR9, zebrafish Nitr9, and catfish IpNITR2, group together suggesting a common ancestry; 2) the majority of the NITRs encoding both V and I domains within a single exon (medaka, Southern pufferfish and *Tetraodon*) group together, suggesting that they are likely derived from a common ancestral gene; and 3) the majority of the NITRs, which lack an I domain (from medaka, zebrafish, catfish, and *Tetraodon*), group together, suggesting that they too may be derived from a common ancestral gene.

**Figure 6:**
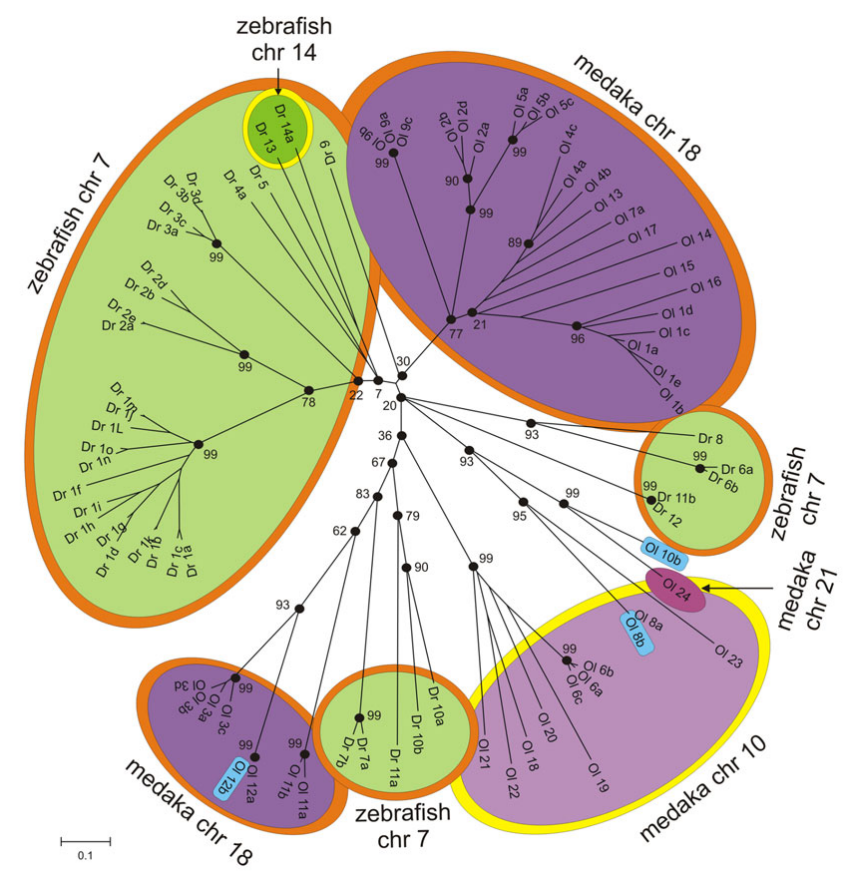
**NITR gene clusters independently evolved in medaka and zebrafish**. Neighbor-joining tree relating NITR V domains in medaka and zebrafish. NITRs that are present within the same gene cluster are highlighted in different shades of purple (medaka) or green (zebrafish). Orange and yellow outlines indicate conserved synteny. Blue indicates genes that are not assigned to a specific chromosome. Branch lengths corresponds to the number of amino acid substitutions estimated by Poisson correction, indicated below. Bootstrap values are indicated at the "major" nodes (black circles).

Multiple species-specific expansions of individual NITR families are revealed by comparing the two complete NITR datasets from medaka and zebrafish (Figure 6). For example, the medaka NITR gene cluster on chromosome 18 includes expansions of the *NITR1*, *NITR2*, *NITR3 *and *NITR5 *families and the corresponding zebrafish NITR gene cluster on chromosome 7 includes expansions of the divergent *nitr1*, *nitr2 *and *nitr3 *families. Three NITRs are encoded by the zebrafish NITR gene cluster on chromosome 14 whereas 10 NITRs are encoded by the corresponding gene cluster in medaka suggesting either gene loss in zebrafish or gene duplications in medaka.

## Discussion

### Interpreting the evolution of NITRs within teleost species

Forty-four different NITR genes, the majority of which are inhibitory, are present in the medaka genome. Perhaps the most striking observation relates to the high degree of variation between these V domains within medaka and their diversification from NITRs in other fish species. This finding is particularly striking given the exceptionally large number of V families in medaka, i.e., the greater the number of V families present, the higher the expectations of identifying orthologous sequences. The medaka/zebrafish chromosomes 18/7 and 10/14, which encode the larger NITR gene clusters, exhibit a high degree of conserved synteny [16]. Medaka chromosomes 10 and 18, and zebrafish chromosomes 7 and 14 likely derive from a hypothetical ancestral chromosome termed "f" which was duplicated prior to the divergence of medaka and zebrafish [16]. Although the nucleotide identity is low between medaka and zebrafish NITR genes (confounding the confident assignment of genetic orthologs), conserved synteny between the two primary medaka and zebrafish NITR gene clusters is impressive.

Comparisons of predicted amino acid sequence structures within and between different species indicate that NITRs are undergoing family-specific expansions and individual genes are undergoing birth and death events. As the NITR gene products are highly diverse within a single species (Figures 3 and 4) and approximately 10% of the NITRs described in medaka can be classified as pseudogenes (Figure 1), it is less likely that concerted evolution played a primary role in establishing the current NITR sequences [19]. Birth and death events of NITR genes likely have been occurring continuously after the divergence of medaka and zebrafish ~323 MYA [16]. Similar observations have been made concerning the V gene segments of the Ig and TCR genes [20] and the KIR genes within primate species [reviewed by [21]]. The KIR gene family is of particular value in comparative interpretations as they also represent type I, Ig domain-containing membrane proteins with potential for inhibitory and activating signalling. KIRs are highly polymorphic and polygenic within single species and drastically diverged in gene sequence and number between different primates [21]. The nature of diversity in KIRs is markedly similar to that observed in the NITRs of medaka and zebrafish. The studies reported here suggest that particularly extensive expansion and diversification of the NITRs have occurred in bony fish. Deducing the status of the NITR gene family from the genomes of more basal fish species (such as gar or sturgeon) will be highly informative regarding the origins of diversity within the NITR family.

### Possible links between NITRs and mammalian receptors

All evidence to date strongly suggests that NITRs are restricted to bony fish. Although NITRs possess authentic V (and frequently V-J) domains reminiscent of TCR, their overall structure, genomic organization and the presence of both activating and inhibitory forms, has led to the hypothesis that they may be functional equivalents to mammalian NK receptors, resembling in some way KIRs. We previously reported that the zebrafish NITR gene cluster on chromosome 7 shares a limited syntenic relationship with the mammalian leukocyte receptor cluster (LRC) that includes the KIRs [14]. The new data presented here introduces two alternative models in which the NITRs share a common ancestry with CD8A/CD8B or with CD28/CTLA4. An expansion of *CD8A*-like genes was recently described within the chicken *CD8A*/*CD8B *locus; the reported structural similarity between the V-like domains of the CD8A-like receptors and NITRs supports the former model [22]. Although intriguing, the latter model is not as well supported. Specifically, in mammals CD28 and CTLA4 effect opposing roles in T-cell stimulation and are encoded by adjacent genes. However, the *CD28 *and *CTLA4 *genes are not physically linked in teleosts. Linkage of an NITR gene to *CTLA4 *has not been identified outside of medaka; in zebrafish the NITR gene clusters are on chromosomes 7 and 14 [2,11]. *CTLA4 *and *CD28 *genes in zebrafish are located on chromosomes 9 and 1, respectively [18].

KIRs, CD8, CD28 and CTLA4 are expressed by cytotoxic lymphocytes and NITRs are similarly expressed in lymphoid populations including NK-like cytotoxic cells [unpublished observations and [3,4]]. In general, inhibitory natural killer cells receptors (NKRs) repress cytotoxicity by recognition of MHC or other markers of "self" on candidate target cells whereas activating NKRs induce cytotoxicity by detecting a loss of "self" or a gain of "non-self" (e.g. viral or stress-related peptides). In at least one case, a NITR specifically engages a non-self determinant (Cannon et al., unpublished observations), suggesting the possibility that they may be involved in cytotoxic interactions. Ongoing efforts to identify NITR ligands and define cell populations that express NITRs *in vivo *will prove crucial in deciphering their role in immunity. In particular, such studies will determine whether their function is analogous to mammalian processes (NK recognition) or represents a unique adaptation within bony fish.

## Conclusion

The medaka genome encodes 44 NITR genes in 3 gene clusters. NITR genes in this species are divergent in sequence but structurally similar to those characterized in other fish species and include both inhibitory and activating forms. Comparative genomics reveals three possible scenarios in which the NITRs share a common genetic ancestry with CD8, CD28/CTLA4 or KIRs. A comparison of the complete sets of NITRs from medaka and zebrafish indicate that: 1) the two largest NITR gene clusters in medaka and zebrafish are derived from a whole chromosome duplication event, 2) as a gene family, the NITRs have experienced recent, species-specific "birth and death" events and 3) NITR family expansions have led to high levels of sequence variation between fish species.

## Methods

### Data mining

Using zebrafish *nitr1c *and *nitr3a *as queries, putative medaka NITR genes were identified by searching version 1.0 of the medaka genome [23]. Manual annotation of these medaka genes was performed using FSPLICE to predict splice sites (GT/AG) and FGENESH+ to predict structures based on homology with zebrafish and other medaka NITR genes: "fish" specific parameters were applied to both programs which are included in the MolQuest software suite [24]. SEQtools was used to visualize the predicted transcripts and their three-frame translations [25]. In addition, V and I domain predictions were validated by searching the NCBI non-redundant database with BLAST [26], while transmembrane predictions were validated using the TMHMM 2.0 server [27]. Lastly, groups of medaka NITRs were defined through BLASTCLUST (single-linkage) based on sharing 70% identity within the peptide sequence of the V-domain. Orthologous genes and their genomic positions were identified in medaka, zebrafish, stickleback, *Xenopus tropicalis*, chicken, mouse and human genomes using ContigView software within the Ensembl genome browser [28].

### Phylogenetic analyses

Published NITR V or I domains were aligned by ClustalW [29] and neighbor-joining trees [30] were constructed from pairwise Poisson correction distances with 2000 bootstrap replications by MEGA2.1 software [31]. NITR sequences described from medaka, zebrafish, Southern pufferfish, channel catfish, rainbow trout, Japanese flounder and *Tetraodon *(Additional file 3) were included in alignments [2-4,10-14].

### Rapid Amplification of cDNA ends

Tissues from young adult (6–9 month), out-bred, orange-red medaka were dissected directly into RNA-Bee and total RNA purified as recommended by the manufacturer (Tel-Test, Friendswood, TX). Two μg of RNA (1 μg from kidney and 1 μg from spleen) were reverse transcribed using the oligo dT primer and Superscript™ III Reverse Transcriptase supplied with the GeneRacer™ kit (Invitrogen, Carlsbad, CA). Nested, forward primers were designed against the exons encoding predicted leader sequences of medaka NITRs and 3' RACE was completed as recommended in the GeneRacer kit. Primer sequences are listed in Additional File 4. PCR products were cloned into pCRII-TOPO (Invitrogen) or pGEM-T Easy (Promega, Madison, WI) and sequenced.

### Accession numbers

Sequence data described in this article have been deposited with the GenBank database under accession numbers: *NITR1*, transcript variant 1 [GenBank:EU419354]; *NITR1*, transcript variant 2 [GenBank:EU419355]; *NITR1*, transcript variant 3 [GenBank:EU419356]; *NITR2*, transcript variant 1 [GenBank:EU419357]; *NITR3*, transcript variant 1 [GenBank:EU419358]; *NITR4*, transcript variant 1 [GenBank:EU419359]; *NITR5*, transcript variant 1 [GenBank:EU419360]; *NITR5*, transcript variant 2 [GenBank:EU419361]; *NITR6*, transcript variant 1 [GenBank:EU419362]; *NITR8*, transcript variant 1 [GenBank:EU419363]; *NITR9*, transcript variant 1 [GenBank:EU419364]; *NITR11*, transcript variant 1 [GenBank:EU419365]; *NITR12*, transcript variant 1 [GenBank:EU419366]; *NITR14*, transcript variant 1 [GenBank:EU419367]; *NITR17*, transcript variant 1 [GenBank:EU419368]; *NITR18*, transcript variant 1 [GenBank:EU419369]; *NITR20*, transcript variant 1 [GenBank:EU419370]; *NITR22*, transcript variant 1 [GenBank:EU419371]; *NITR24*, transcript variant 1 [GenBank:EU419372]; *NITR24*, transcript variant 2 [GenBank:EU419373]; and *NITR24*, transcript variant 3 [GenBank:EU419374].

## Authors' contributions

SD completed the gene prediction analyses. AKH, TMO and JAY cloned and sequenced the medaka NITR cDNAs. JAY conducted the phylogenetic analyses and comparative genomics. GWL and JAY interpreted data and wrote the manuscript. All authors have read and approved the final version of the manuscript.

## Supplementary Material

Additional File 1 Predicted nucleotide sequence of NITRs based on the 1st draft of the medaka Hd-rR genome. Sequences are in FASTA format and include only full-length open reading frames. Predicted exons are color-coded (leader = green; V domain = light blue; I domain = red; transmembrane domain = pink; cytoplasmic exons are dark blue and yellow). Click here for file

Additional File 2 Protein sequences encoded by predicted NITR transcripts based on the 1st draft of the medaka Hd-rR genome. Sequences are in FASTA format and include translated sequences from Additional file 1. Predicted protein domains are color-coded (leader = green; V domain = light blue; I domain = red; transmembrane domain = pink; cytoplasmic domain = yellow). Click here for file

Additional file 3 *Tetraodon nigroviridis *encode multiple NITRs. Putative NITR genes and gene transcripts were identified from *Tetraodon nigroviridis *by a tBLASTn search using a *Spheroides nephelus *NITR1 protein sequence [GenBank:AAF28781] as a query. This strategy identified six candidate *Tetraodon *cDNAs [GenBank:CR683680, GenBank:CR675622, GenBank:CR664265, GenBank:CR725053, GenBank:CR724466, and GenBank:CR707240] and a single genomic BAC clone B22K12 [GenBank:BX629355]. Only one of these cDNAs [GenBank:CR683680] appears to include an entire ORF. The partial protein sequences encoded by [GenBank:CR675622] and [GenBank:CR664265] are identical. The partial protein sequences encoded by [GenBank:CR725053] and [GenBank:CR724466] differ at three predicted amino acids residues; [GenBank:CR725053] is 10 amino acids longer at the amino-terminus. BAC B22K12 encodes 12 different NITR genes (designated by their nucleotide location in the reverse complement position within BAC B22K12). The predicted protein sequences of four representative NITR cDNAs are aligned relative to the predicted protein sequences of the NITRs encoded by BAC B22K12. Note that NITR-116851 (BAC B22K12) is predicted to encode a V domain and lacks an I domain. Click here for file

Additional File 4 Oligonucleotide primer sequences used for nested 3' RACE. Predicted translational start sites are underlined. Primers designed to amplify *NITR10 *amplified *NITR24 *transcripts. Primers designed to amplify *NITR13 *amplified *NITR4 *transcripts. Primers designed to amplify *NITR16 *amplified *NITR1 *transcripts. Click here for file

Additional File 5 Classification of medaka NITR cDNAs. Neighbor-joining tree of V domains encoded by out-bred, orange-red medaka NITR cDNAs (highlighted in yellow) and predicted NITR V domains from the genome of the inbred Hd-rR medaka. The number assigned to each interior branch corresponds to the bootstrap value. Branch lengths correspond to the number of amino acid substitutions estimated by Poisson correction, scale indicated below. Click here for file

Additional File 6 Variation between the medaka *NITR12 *cDNA and genomic sequence. A partial alignment of *NITR12 *cDNA from out-bred, orange-red medaka to the predicted coding sequence of the *NITR12a *gene from inbred Hd-rR medaka. Predicted peptide sequence is shown above (cDNA) and below (genomic DNA) the alignment. The sequences shown include a portion of the transmembrane domain exon and the two cytoplasmic domain exons. Dashes indicate gaps in the nucleotide alignment. Asterisks indicate nucleotide identity. Stop codons are indicated by red highlighting in the nucleotide sequence and by dashes in the protein sequence. Downward arrowheads define predicted RNA splice sites. The *NITR12 *cDNA encodes additional nucleotide sequences that are not present in the genomic sequence (nucleotides highlighted in pink). Although the *NITR12a *genomic sequence is predicted to encode an ITIM (highlighted in yellow) and a second ITIM-like sequence (itim; highlighted in orange), differences in the *NITR12 *cDNA alter the predicted ITIM sequence (LNYAAL to RYYAAL) and introduce a stop codon, preventing translation of the itim. The differences in predicted protein sequence likely reflect allelic variations between the out-bred, orange-red transcripts and Hd-rR genomic sequence. Click here for file

Additional File 7 Joining (J) and J-like sequences in medaka NITRs. The consensus joining (J) sequence is FGXGTXLX(V/L). A J-like sequence possesses either motif FGXG or TXLX(V/L/I) or partial sequence of both motifs. Sequences were compiled from version 1.0 of the Hd-rR genomic sequence. Click here for file

Additional File 8 ITIM and ITIM-like sequences in medaka NITRs. Consensus ITIMs are defined as (S/I/V/L)xYxx(I/V/L) [33]. An ITIM-like sequence lacks a consensus residue in either the first or last position. Sequences were compiled from version 1.0 of the Hd-rR genomic sequence. Click here for file

Additional File 9 Transmembrane domains of activating NITRs. Transmembrane domains predicted by SMART software [34]. Charged residues are white text on black. Zebrafish Nitr9 has been shown to partner with and signal via Dap12 [15]. Catfish IpNITR2, IpNITR3, IpNITR4, IpNITR10 and IpNITR11 encode transmembrane domains similar to medaka NITR9 and zebrafish Nitr9 [4]. Click here for file

Additional File 10 The *CD8 *locus does not display conserved synteny between medaka, zebrafish, stickleback and human. Although the *CD8A *and *CD8B *genes are tightly linked in **(a) **medaka, **(b) **zebrafish, **(c) **stickleback and **(d) **human, flanking genes are not well conserved between fish and human. Data were acquired using release NCBI 36 of the human genome, release 1.0 of the medaka genome, release Zv7 of the zebrafish genome and release 1.0 of the stickleback genome. Click here for file

Additional File 11 Medaka *NITR24 *is linked to *CTLA4*. **(a) **Human *CTLA4 *and *CD28 *are tightly linked. **(b) **In medaka *CTLA4 *is closely linked to *NITR24*. Gene names and chromosomal order were identified via Contig View within the Ensembl Genome Browser [28]. Three predicted olfactory receptor (OR) genes are indicated on medaka chromosome 21, although the exact number of OR genes has not been determined. Data were acquired using release NCBI 36 of the human genome and release 1.0 of the medaka genome. *CTLA4 *and *CD28 *are not linked to *NITR *genes in zebrafish (not shown). Note that Ensembl misidentified medaka *CTLA4 *[18] as *CD28*. Predicted protein structures are shown and structural features are as in Figure 4 plus cytoplasmic activating motifs (YxxM); and a functionally unresolved motif (YxxH). Click here for file

Additional File 12 Phylogenetic comparisons of published NITR V domains. **(a) **Cladogram depicts conventional phylogenetic radiations of bony fish [Figure adapted from 35]. Colors denote species in which NITR genes and/or gene transcripts have been characterized. **(b) **Neighbor-joining tree of V domains encoded by 73 representative NITR genes. Ol, medaka (*Oryzias latipes*); Dr, zebrafish (*Danio rerio*); Ip, channel catfish (*Ictalurus punctatus*); Om, rainbow trout (*Oncorhynchus mykiss*); Sn, Southern pufferfish (*Spheroides nephelus*); Po Japanese flounder (*Paralichthys olivaceus*) and Tn, pufferfish (*Tetraodon nigroviridis*). Specific NITR gene numbers are indicated (e.g. medaka NITR8a = Ol 8a), except for *Tetraodon *sequences (see Additional file 3). Only one NITR gene family member is incorporated; additional family members are indicated in parenthesis. Activating NITR genes are indicated by a "+"; NITR families that possess both inhibitory and activating forms are indicated by a "+/-". NITR genes which encode a V and I domain in a single exon ("V and I exons fused") and "No I domain" are indicated. These labels are presented in parentheses for NITR families, which do not consistently conform to these definitions. Number assigned to each interior branch corresponds to the bootstrap value; bootstrap values less than 60 are not shown. Branch lengths correspond to number of amino acid substitutions estimated by Poisson correction, indicated below. Click here for file
