# Supplementary material for: The medaka novel immune-type receptor (NITR) gene clusters reveal an extraordinary degree of divergence in variable domains
Source: BMC Evol Biol. 2008 Jun 19;8:177. doi: 10.1186/1471-2148-8-177 (PMC2442602; doi:10.1186/1471-2148-8-177)
Supplement: Additional File 1 — Predicted nucleotide sequence of NITRs based on the 1st draft of the medaka Hd-rR genome. Sequences are in FASTA format and include only full-length open reading frames. Predicted exons are color-coded (leader = green; V domain = light blue; I domain = red; transmembrane domain = pink; cytoplasmic exons are dark blue and yellow). [file 1471-2148-8-177-S1.doc]

>NITR1a

ATGGTGACACCTGCTCAGTTTGTCGTCATGCTGACATGTTTGCTTTCAGGGAACTCTGCACAGAGCCCTCCTCTGGGATCATCTTCATCTGATCACCAAGAAAGTGTTTTTCTATCAGCTCACATTGGAGAAACTGTGACTTTACAATGTTTCTATGATGGAGTTTTTCTGCAATATATTTTATGGTACAAACATATTCTGGGACGAAAACCAAAACTTATCGCATATTTCACCAAATATGAAACACCATTAAAAATTTCCCATGAATACCAGAACAATCCACGCTTTACACTAAAATCATCACAACAAAGTAGTAATTTGACAATTTCAGATCTGAAACCATCAGACTCAGCTACTTACTTCTGCATTTCAGATCATCAAACATATTTGTTTTTTAAATCAGCTTTTACACTTGATGTAAAAGGTTCAGGTTTAACCATCCAGACTTCAGTGAGTCAGTCGTCCTCTGAGAACATCCATGCAGGAGACTCTGTGACTCTGAACTGTACAGTACACACTGGGAGCTGTGATGAAGAACACAGAGTTTACTGGTTCAAAGACTCTGAAGACTCTCATCCAGGACTCATTTACACTCATGGAGGCAGGAATGATCAGTGTGAGAGAAAGAACAACACACAAACACACAGCTGTGTCTATGAGCTGCACATAAAGAACCTGACAGAGTCTCATGCTGGGATCTACTACTGTGCTGTTGTCTCATGTGGACACATACTGTTTGGAAACGGAACCAAGCTGGACCTCACAGCTCCTCTGAACTCTGTGATTCTGAACACTTTGGTTGGATTATTGACGGTCATGAGCGTCCTCGTTGTTTTTCTGCTTTTTCTGCTGTGGAAAATCCACAAGAGCAACAACTGCACCTCCACAGAGGAAAGATCACCTGCTGCCCCCTTAAGAAGCTCAGAGGCTGAGAACAAAGACACAGAAAGCCTCCATTATGCTGCTGTTAATGTTAAGAAGTCCAACAGATCAAGGAGACAGAAGAACGAAGCCAAAACCGACTATCTGTATGCGAGTGTGAGACAGCAGAACTAA

>NITR1b

ATGGTGACACCTGCTCAGTTTGTCGTCATTCTGACATGTTTGCTTTCAGGGAACTCTGCACAGAGCCCTCCTCTGGGATCATCTTCATCTGATCACCAAGAAAGTGCTTTTCTATCAGCTCACATTGGAGAAACTGTGACTCTACCATGTTTCTATGACGGAATTTATTTGCGATATATTTTATGGTACAAACATATTCTGGGACGAAACCCAACATTTATTGCATATTTCACCAAATATAGTCCAGAAAAAGAAATTTCCCATGAATACCAGAACAATCCACGCTTTACACTAAAAACATCAGAACAAAGTAGTAATTTGACAATTTCAGATCTGAACCTATCCTACTCCGCTACGTACTTCTGCATTTCACATCATTATACGTATTTGTTTTCTCAAGCAGCTTTTACACTTGATGTAAAAGGTTCAGGTTCAACCATCCAGACTTCAGTGAGTCAGTCGTCCTCTGAGAACATCCATGCAGGAGACTCTGTGACTCTGAACTGTACAGTACACACTGGGAGCTGTGATGAAGAACACAGAGTTTACTGGTTCAAAGACTCTGAAGACTCTCATCCAGGACTCATTTACACTCATGGAGGCAGGAATGATCAGTGTGAGAGAAAGAACAACACACAAACACACAACTGTGTCTACAAGCTGTCCATAAAGAACCTGACAGAGTCTCATAATGGGATCTACTTCTGTGCTGTTGTCTCATGTGGACACATACTGTTTGGAAACGGAACCAAGCTGGACCTCACAGACTCTGCAGCTCCTCTGAACTCTGTGATTCTGAACACTTTGGTTGGATTATTGACGGTCATGAGCGTCCTCGTTGCTTTTCTGCTTTTTCTGCTGTGGAAAATCCACAAGAGCAACAACTGCACCTCCACAGAGGAAAGATCACCTGCTGCCCCCTTAAGAAGCACAGAGGCTGAGAACAAAGACGCAGAAAGCCTCCATTATGCTGCTGTTAATGTTAAGAAGTCCAACAGATCAAGAAGACAGAATGATAACTCCAACAACGACTGTCTGTATGCGAGTGTGAGACAGCAGAACTGA

>NITR1c

ATGGTGACACCTGCTCAGTTTGTGGTCTTACTGACATGTTTGCTTTCAGGGAACACTGCACAGAGCCCTCTTCTGGGATCATCTTCATCTGATCACCAAGAAAGTGTTTTTCTATCAGCTCACATTGGAGAAACTGTGACTTTACCATGTTTCTATGACAGAGTTTATTTCCGATATATTTTATGGTACAAACATATTCTGGGACAAAAACCAAAACTTGTAGCATATTTTACCAAATATAATCCAGATATACCAATTCCCCATGAATACCAGAACAATCCACGCTTTACACTAAAAACATCAGACCAAAGTAGTAATTTGACAATTTCAGATCTGAACCTATCAGACTCTGCTACTTACTTCTGTATTTCAGATCATCTAACACACATGAATTTCTCAGCAGTTCTTACCGTCAATGTAAAAGGTTCAGGTTTAACCATCCAGACTTCAGTGGATCAGTCTTCCTCTGAGAACATCCATGCAGGAGACTCTGTGACTCTGAACTGTACAGTACACACTGGGAGCTGTGATGAAGAACACAGAGTTTACTGGTTCAAAGACTCTGAAGACTCTCATCCAGGACTCATTTACACTCATGGAGGCAGGAATGATCAGTGTGAGAGAAAGAACAACACACAAACACACAGCTGTGTCTACAAGCTGCACATGAAGAACCTGACAGAGTCTCATGCTGGGATCTACTACTGTGCTGTTGTCTCATGTGGACACATACTGTTTGGAAACGGGACCAAGCTGGACCTCACAGACTCTGCAGCTCCTCTGAACTCTGTGATTCTGAACACTTTGGTTGGATTATTGACGGTCATGAGCGTCCTCGTTGCTTTTCTGCTTTTTCTGCTGTCGAAAATCAACAAGAGCAACAACTGCACCTCCACAGAGGAAAGATCACCTGCTGCCCCCTTAAGAAGCACAGAGGCTGAGAACAAAGACGCAGAAAGCCTCCATTATGCTGCTGTTAATGTTAAGAAGTCCAACAGATCAAGAAGACAGAATGATAACTCCAACAACGACTGTCTGTATGCGAGTGTGAGACAGCAGAACTGA

>NITR1d

ATGGGGACACCTGCTCAGTTTGTGGTCATTCTGACATGTTTGCTTTCAGGGAACACTGCACAGAGCCCTCCACTGGGATCATCTTCATCTGATCACCAAAAAAGTGTTTTTCTATCAGCTCACATTGGAGAAACTGTGACTTTACAATGTTTCTATGACGGAGTTTATTTGCAATATATTCTATGGTACAAACATATCCTGGGACGCAAACCAAAGCCTATCTCTCTTTTCAGTAAATATAGTGCAGAGTTAATTTATTACAATAATTATAAGAACAATCCACGCTTTACTTTAACAACATCAGACCAAAGCATCAGTTTGATCATTTCAAATCTGAAACAATCAGACTCAGCAATTTACTTCTGTGTGGCTGGTTATCAAACACACATGAATTTCTCTGCAGTTCTTACAGTCAATGTAAAAGGTTCAGGTTTAACCATCCAGGCTTCAGTGGATCAGTCGTCCTCTGAGAACATCCATGCAGGAGACTCTGTGACTCTGAACTGTACAGTCCACACTGGGAGCTGTGATGAAGAACACAGAGTTTACTGGTTCAAAGACTCTGAAGACTCTCATCCAGGACTCATTTACACTCATGGAGGCAGGAATGATCAGTGTGAGAGAAAGAACAACACACAAACACACAGCTGTGTCTTCAAGCTGCACATAAAGAACCTGACAGAGTCTCATGCTGGGATCTACTACTGTGCTGTTGTCTCATGTGGACACATACTGTTTGGAAACGGGACCAAGCTGGACCTCACAGGCTCTGCAGTTCCTGTGAACTCTGTGATTCTGAACACTTTGGTTGGATTATTGACGGTCATGAGCGTCCTCGTTGTTTTTCTGCTTTTTCTGCTGTGGAAAATCAACAAGAGCAACAACTGCACCTCCACAGAGGAAAGATCACCTGCTGCCCCCTTAAGAAGCACAGAGGCTGAGACAAAAGACACAGAAAGCCTCCATTATGCTGCTGTTAATGTTAAGAAGTCCAACAGATCAAGAAGACAGAAGAACGACTCCAACACCGTCTGTGTTTATGCGAGTGTGAGACAGCAGAACTGA

>NITR2a

ATGACTCTTGTCGTGTTTGCTGGCTGTGTGACATGTCTGCTCCTGGGAACAGTGGCTAATTCTTGGGCTCAGGAATCGTCTGCTTCTTTACATTTTGAATCAGTTTTTGTTGGACAAGAAGTGACCTTAAAATGTTTTCTTGAAGGCACTGGGGCAAATGTCTTTTTCTGGTATAAACAACCTCTGGGACAGAAACCACAACTCATGTCTGAATTTTTCAATCATAAGGAAATTGGAACTTTTGCAGATGATTTCAAGAAGGATCCACGGTTTGAACTGCAGACAAATGAAGACAAAAACAACTTGAAGATCTCAAATGTGAAAATGTCAGATTCTGCTACGTACTACTGCATCAGTTCTTACACGTATACACTCACATTTTTGGAGGCCTATAGTCTCCATGTGAGGGACACGTCGTCTGACATCCAGACTTCAGTGGATCAGTCGTCCTCTGAGAACATCCATGCAGGAGACTCTGTGACTCTGAACTGTACAGTACACACTGGGAGCTGTGATGAAGAACACAGAGTTTACTGGTTCAAAGACTCTGAAGACTCTCATCCAGGACTCATTTACACTCATGGAGGCAGGAATGATCAGTGTGAGAGAAAGAACAACACACAAACACACAGCTGTTTCTACAAGCTGTCCATAAAGAACCTGACAGAGTCTCATGCTGGGATCTACTACTGTGCTGTTGTCTCATGTGGACACATACTGTTTGGAAACGGGACCAAGCTGGACCTCACAGCACAAGAAGGGAATCTTCTTCCCTTTGTGTATTTCCTGAGTGGAGCTCTGACAGCTTCGTTGATCTTCCTCACATCTTACGCAATACACAAGACAAAGAGCTGCAAATCCAAGGGCGTCTGGGGCGTCAAGGTGCCAAATACATTTTTGGATAGGCATCAAGCAGTAAATTTCACGAGCGCCAAATGTGAATTTGACGTACGAATCGCCCTCGATGTGAATCCAGCTTTATGGATCAATTCCAATAATATTTTGTGCTACGTTCAAAGAGCTTCCAGTGGTCTTTTAGAAATAAGGATGCTGTTCTAA

>NITR2b

ATGACTCTTCTGGTGTTTGCTGGCTGTGTGACATGTCTGCTCCTGGGAACCGTGGCTAATTCTTGGGCTCAGAAATCGTCTGCTTCTTTACATTTTGAATCAGTTTTTGTTGGACAAGAAGTGACTTTAAAATGTTTTCATGGAGGCACTGTGGCAGATTTCTTTTTTTGGTATAAACAACCTCTGGGACAAAAACCACAGCGCATGTCTACATTTTTGGATTATAACAAAAATGGAACTTTTTTGGATGATTTCAAGAAGGATCTACGGTTTGAACTACAGACAAATAAAGACACACACCACTTGAAGATCTCAAATGTAAAAATGTCAGATTCTGCTACCTACTACTGCATCAGTTCTTATTCTTATGCATTTACATTTTTGGAGGCCTATAGTCTCCATGTGAAGGACCATTCTTACATCCAGACTTCAATGGATCAGTCGTCCTCTCAGAACATCCATGCAGGAGACTCTGTGACTCTGAACTGTACAGTACACACTGGGAGCTGTGATGAAGAACACAGAGTTTACTGGTTCAAAGACTCTGAAGACTCTCATCCAGGACTCATTTACACTCATGGAGGCAGGAATGATCAGTGTGAGAGAAAGAACAACACACAAACACACAGCTGTGTCTATGAGCTGCCCATAAAGAACCTGACAGAGTCTCATGCTGGGATCTACTACTGTGCTGTTGTCTCATGTGGACACATACTGTTTGGAAACGGGACCAAACTGGAACTCACAGGTCAGACAAACTCTTTGTTGGTGTATTTCTTAACTGGAACTTTGACCTTCATGAGCATCCTTGTTGTTTTTCTGGTTTACAAGATTAAAAAACACATTTGCTTCCAGTCTAAAGATGAGGGGTCAGCAGCTGCCTCTACACCAAATACAGAGGCTGAGAACAAAGACACAGAAAGCCTCCATTATGCTGCTGTTAATGTTAAGAAGTCCAACAGATCAAGAAGACAGAAGAACGACTTAAACACAGTCTGTGTTTATGCCAGTGTGACACAACAGAACTGA

>NITR2d

ATGACTCTTCTGGTGTTTGCTGGCTGTGTGACATGTCTGCTCCTGGGAACAGTGGCTTATTCTTGGGCTCAGAAATCTTCTGCTTCTTTACATTTTGAATCAGTTCTTGTTGGACAAGAAGTGACTTTGAAATGTTTTCATAGAGGGACTGTAGCAGATTTTTATTTTTGGTATAAACAACCTCTGGGACAGAAACCACAGCGCATGTCGGAATTTTTCGATTATAAGAAAAATGGAACTTTTTCAGATGATTTCAAGAACGATCTACGGTTACAACTACAGACAAATGAAGGCAAAAACCACTTGAAGATCTCAAATGTGAAAATGTCAGATTCTGCTACTTACTACTGCATCAGTTCTTACACTTATACATTCACATTTTTGGAGGCCTATAGTCTCCATGTGAGGGACACGTCGTCTGACATCCAGACTTTGGTGACTCAGTCGTCCTCTGAGAACATCCATGCAGGAGACTCTGTGACTCTGAACTGTACAGTCCACACTGGGAGCTGTGATGAAGAACACAGAGTTTACTGGTTCAAAGACTCTGAAGACTCTCATCCAGGACTCATTTACACTCATGGAGGCAGGAATGATCAGTGTGAGAGAAAGAACAACACACAAACACACAGCTGTGTCTATGAGCTGCACATGAACAACCTGCATGAGCCTGGGATCTACTACTGTGCTGTTGTCTCATGTGGACACATACTGTTTGGAAACGGAACCAAGCTGGACCTCACAGGTCAGACAAACTCTTTGTTGGTGTATTTCTTAACTGGATCTTTGACCTTCATGAGCATCCTTGTTGTTTTTCTGGTTTACAAGATAAAAAGACAAATTTGCTTCCAGTCTAAAGATGAAGGGTCAGCAGCTGCCTCTACACCACAAACAGAGGCTGAGAACAAAGACACAGAAAGCCTCCATTATGCTGCTGTTAATGTTAAGAAGTCCAACAGATCAAGAAGACAGAAGAACGGAGGCAACACCGACTGTGTTTATGCCAGTGTGAGACCGCAGAACTGA

>NITR3a

ATGGTGAACCTTACCTTGGTTCTGGTTTTGCTCTGCACCCTCAGTCTGATCTCTTTGTCAACCCCTGAGTTTCACACAGTGAAAGTTCAACCTGAAGGAGAAGTTACACTGAAGTGCTCAAACTTCAGCAACTTCATATCTTACATTGTCTGGTTCAAACTGAATGATGGACGCAACGCCACAATCATTTCATCAATGATAACCTCAGAGTCCAATGTTTCAATGAAGGATGGATTCAAAGAAAGATTTTTTATGACATCTAACATCACACATGTTTTTCTCAACATCAAAAATGTGAACCTCTCTGACTCTGGACTATATTTCTGTGGACATAGAGGCAGTACAAGCGCAGTTATTTTTGGTGCAACGTATTTGCTGGTATATGAAATGTCCAGATCACCAGACCTTCAGACCGTGATTGTGGGAAGCATTATTGCTTTTCTTCTGATGGTCATCATCATGCTGTTCATGAAAATCCGGTCTTATCCAAAAGCTTCAGTTGAAAGACAACTAAAAGAGAGCCTGGACTCTGATGCTTTAAACTATGCAGCTGTAAGTTTCCAATGGAAAGCAAAGACCAGGATAAATGCTGCATACACAAAGGTGGTTTATGCAACCACTAAATAG

>NITR3b

ATGGTGAACCTTACCTTGGTTCTGGCTTTACTCTGCACCCTCAGTCTGATCTCTTTGTCAACCCCTGAGTTTCACACAGCAAAGGTTCAACCTGAAGGAGAAGTTACACTGAAGTGCTCAAACTTCAGCAACTTAATATCTAACATTCTCTGGTTCAAACTGAATGATGGACGCAACGCCACAATCATTTCATCAATGATAACCTCAGAGTCCAACGCTACTATGTTGGATGGATTCAAAGAAAGATTTTTTATGACATCTAACATCACACATGTTTTTCTCAACATCAAAAATGTGAACTTCTCTGACTCTGGACTATATTTCTGTGGACATAGAGGCAGTACAAGCGCAGTTATTTTTGGTGCAACGCATTTGCTGGTATATGAAATGTCCAGATCACCAGAACTGCTGACCATAATTCTGGGAAGCATTATTGCTTTTCTTCTGATGGTCATCATCGTCATGCTGTTCATGAAAATCAGGTCTTATCCAAAAGCTCCAGTTGAAAGACACCAAAAAGAGAACCTGGACTCTGATGCTTTGAACTATGCAGCTGTCAGTTTCCGATGGAAAGCAAAGACCAGGACAAAAGCTGCAGAAGACATAAATGTGGTTTATGCAACCACTAAATAG

>NITR3c

ATGGTGAACCTTACCTTGGTTCTGGCTTTACTCTGCACCCTCAGTCTGATCTCTTTGTCAACCCCTGAGTTTCACACTTTGAAAGTTCAACTTGAAGGAGAAGTTACACTGAAGTGCTCAAACTTCAGCGACTTAATATCTAACATTCTCTGGTTCAAACTGAATGATGGACCCAATGCCACAATCATTTCATCAATGATAACCTCAGACTCCAACGCTTCAATGAAGGATGGATTCAAAGAAAGATTTTTTATGACATCTAACATCACACATGTTTTTCTCAACATCAAAAATGTGAACTTCTCTGACTCTGGACTATATTTCTGTGGACATAAAGATGGTGCAAGAGCAGTTATTTTTGGTGCAACGTATTTGCTGGTTAATGAAATGTCCAGATCAGCAGAACTGCAGACCATAATTCTGGGAAGCATTATTGCTTTTCTTCTGATGATCATCATCGTCATGCTGTTCATGAAAATCAGGTCTTATCCAAAAGCTCCAGTTGAAAGACAACAAAAAGAGAACCTGGACTCTGATGCTTTGAACTATGCAGCCGTCAGTTTCCGACGGGAAGCAAAGAACAGGACAAAAGCTGCAGAAGACATAAACGTGGTTTATGCAACCACTAAATAG

>NITR3d

ATGGTGAACCTTACCTTGGTTCTGGTTTTGCTCTGCACCCTCAGTCTGATCTCTTTGTCAACCCCTGAGTTTCACACAGCAAAGGTTCAACCTGAAGGAGAAGTTACACTGAAGTGCTCAAACTTCAGCAACTTAATATCTAACATTCTCTGGTTCAAACTGAATGATGGACGCAACGCCACAATCATTTCATCAATGATAACCTCAGACTCCAACGCTTCAATGAAGGATGGATTCAAAGAAAGATTTTTTATGACATCTAACATCACACATGTTTTTCTCAACATCAAAAATGTGAACTTCTCTGACTCTGGACTGTATTTCTGTGGACATAGAGGCAGTACAAGCGCAGTTATTTTTGGTGCAACGCATTTGCTGGTATATGAAATGTCCAGATCACCAAACCTTCAGACCATAATTCTGGGAAGCATTATTGCTTTTCTTCTGATGGTCATCATCGTCATGCTGTTCATGAAAATCAGGTCTTATCCAAAAGCTCCAGTTGAAAGACAACAAAAAGCGAACCTGGACTCTGATGCTTTGAACTATGCAGCTGTCAGTTTCCGACGGAAAGCAAAGACCAGGACAAAAGCTGCAGAAGACATAAACGTGGTTTCAGTCAGACAACTCTTGCAGTAA

>NITR4a

ATGATCTCGAAGCGCTTTGTTTTCTTTCTGACACTTTTGTTTGTAGCAGCAAATGGACAGAAGACGTATCCGAAGGCATCCTCCTCTGTACAGCAGAAAGGCAAAGGTTTCCTTTCTGTCTCGGTTGGTGACACTGTTACTTTAGAATGTTCTTACGAAGGTCCTAATTCAGTTCAGATCTTCTGGTACAAGCAAAGTTTTGGACAAAAGCCCAAGCTTATGTCCACTTTTTATGGGCACACTACAAAGGAGACCTTTTCTGATGAATTTAAGAGCAATTCACGTTTCAAACTGGAGACAGAAAACCAAAATCCTCACTTGAAGATTTCTAATTTAAAATTTTCAGACTCTGCTACCTACCACTGCATAAGTTCTGATTCTTACTCACTTACTTTTTTGGAGAGCTTTCGTGTCCTTGTGAAGGACCCTTCTTCTTATGTTCTGACTTCAGTGGATCAGTCGTCCTCTGAGAACATCCATGCAGGAGACTCTGTGACTCTGAACTGTACAGTACACACTGGGAGCTGTGATGAAGAACACAGAGTTTACTGGTTCAAAGACTCTGAAGACTCTCATCCAGGACTCATTTACACTCATGGAGGCAGGAATGATCAGTGTGACAGAAAGAACAACACACAAACACACAGCTGTGTCTATGAGCTGCACATAAAGAACCTGACAGAGTCTCATGCTGGGATCTACTACTGTGCTGTTGTCTCATGTGGACACATACTGTTTGGGAACGGGACCAAGCTGGACCTCACAGTTCCTCTGAACTCTGTGATTCTGAACACTTTGGTTGGATTATTGACGGTCATGAGCGTCCTCGTTGCTTTTCTGCTTTTTCTGCTGTGGAAAATCCACAAGAGCAACAACTGCACCTCCACAGAGGAAAGATCACCTGCTGCCCCCTTAAGAAGCTCAGAGGCTGAGAACAAAGACGCAGAAAGCCTCCATTATGCTGCTGTTAATGTTAAGAAGTCCAGCAAAACAAGAAGACAGAAGAACAACTCCAACACAGTCTGTGTTTATGCGAGTGTGAGACAGCAGAACTGA

>NITR4b

ATGATTTCTAAGCTTTTTGTTTTCTTTCTGACATGTGTGTTCATAGAAGCAAATGGACAGAAGACTCATTTAAAATTGTCCTCTTCTGAAGAGCAGAAACAATTTTTTTTATCTGTTGATGCAGGTGAAACTGTCACTTTAAAATGTTCTTATGAGGGTCATACTTTACGATGGATATTCTGGTACAAGCAAAGTTTGGGACAGAAGCCTAAACTTCTGTCAAGTTTCTATGTGCACGGCACAGAAGTGATCTTTTTTGATGAGTTCAAGAACAATTCACGCTTCAAATTGGATACAGAAAACAAAAATTTTCACTTGAAAATTTCTAATTTACAATTTTCAGATACGGCTACCTACCACTGCATAGGTTCTGATTCTTACTCACTTACATTTGTCGAGGGTTATAGTGTCCATGTGAAGGACCGATCTTTTTACCTCAAGTTTTCCTCTGAGAACATCCATGCAGGAGACTCTGTGACTCTGAACTGTACAGTACACACTGGGAGCTGTGATGAAGAACACAGAGTTTACTGGTTCAAAGACTCTGAAGTCTCTCATCCAGGACTCATTTACACTCATGGAGGCAGGAATGATCAGTGTGAGAGAAAGAAAAACTCACAAACACACAGCTGTGTCTATGAGCTGTACATGAAGAACCTGACAGAGTCTCATGCTGGGATCTACTACTGTGCTGTTGTCTCATGTGGACACATACTGTTTGGAAACGGGACCAAGCTGGACCTCACAGCTCCTCTGAACTATGTGAATCTGAACACTTTGGTTGGATTATTGACGGTCATGAGCGTCCTCGTTGCTTTTCTGTTTTTTCTGCTGTGGAAAATCCACAAAAGCAACAACTGCACATCCACAGAGGAAAGATCACCTGCTGCCCCCTTAAGAAGCTCAGAGGCTGAGAACAAAGACACAGAAAGCCTCCATTATGCTGCTGTTAATGTTAAGAAGTCCAGCAGATCAAGAAGACAGAAGAACGACTTAAACACAGTCTGTCTTTATGCGAGTGTGAGACAGCAGAACTGA

>NITR4c

ATGATTTCTAAGCTCTTTGTTTTCTTTCTGACATGCTTGTTTGTAGGAGGAAATGGTCTAGACATGTATCCAAAAATATTATCTTCTGTACAGCAGAAAGGAGATTTTCTATCTGCCTACGTTGGTGAAACTGTTACTTTAGAATGTTCTTATGAAGTTAAAACTGTAAAACGGATTTTCTGGTCCAAGCAAAGATTACGACGCAAACCTGAACTTCTGTCAAGTTTCTATGTGCACAGCACAGAAGTGACATTTTTTGATGAGTTCAAGAACAAATCACGCTTTAGACTGGATACAGAAAACCAAAATCATCACCTGAAGATTTTGAATTTACAATTTTCAGACTCGGCTACCTATAACTGCATAAGTTCTGATTCTTACTCACTTACATTTTTGGAGAGCTTTAGTGTCCTTGTGAAGGACCCTTCTTCTTACGTTCTGACTTCAGTGAGTCAGTCATCCTCTGAGAACATCCATGCAGGAGACTCTGTGACTCTGAACTGTACAGTACACACTGGGAGCTGTGATGAAGAACACAGAGTTTACTGGTTCAAAGACTCTGAAGACTCTCATCCAGGACTCATTTACACTCATGGAGGCAGGAATGATCAGTGTGAGAGAAAGAACAACACACAAACACACAGCTGTGTCTATGAGCTGCACATGAAGAACCTGACAGAGTCTCATGCTGGGATCTACTACTGTGCTGTTGTCTCATGTGGACACATACTGTTTGGAAATGGGACCAAGGTGGACCTCACAGACTCTGCAGCTCCTCTGAACTCTGTGATTCTGAACACTTTGGTTGGATTATTGACGGTCATGAGCGTCCTCGTTGCTTTTCTGCTTTTTCTGCTGTGGAAAATCCACAAAAGCAACAACTGCACCTCCACAGAGGAAAGATCACCTGCTGCCCCCTTAAGAAGCTCAGAGGCTGAGAACAGAGACGCAGAAAGCCTCCATTATGCTGCTGTTAATGTTAAGAAGTCCAACAGATCAAGAAGACAGAAGAACGACATAAACACAGTCTGTCTTTATGCCAGTGTGAGACAGCAGAACTGA

>NITR5a

ATGACTCTTCTGGTGTTTTCTGGCTGTCTGACATGTCTGCTGCTGGGAACAGTGGCTCTTTCTTGGGCTCAGAAACCGTCTGCATCTTTACAGTTTCAATCTGTTCATGTTGGTGATGAAGTGACTTTAAAATGCATTCGTCAAGGTACTGGGATAGAGAATATTTATTGGTATAAACAACCTCTAGGAATGAAACCACAACTCATGTCTGAGTATTTAGATATCAAGAAAAATGGATATTTTATAGATGCTTTCAAGAACGATCCACGGCTTAAACTGGAAACAGACAAAAACAAACACCACCTGAAGATCTCAAATTTGAAAATGTCAGACTCCGCCACCTACTACTGCATCAGTTCTGATTTTTATGGGGTAAAACATCTGGAGGGCTATACTGTCCATGTGAAGGACTCTACTTCTGACATTCATGCTTCAGTGGATCAGTCGTCCTCTGAGAACTTCCATGCTGGACACTCTGTGACTCTGAACTGTACAGTACACACTGGGAGCTGTGATGGAGAACGGCAAGTTTACTGGTTCAAAGACTCTGGAGACTCTCATCCAGGACTCATTTACACTCATGGAGGCAGGAATGATCCGTGTGGGAGAAAGAACAACACACAAACACACAGCTGTGTCTACGAGCTGCACATGGAGAGGCTGACTGAGTCTCATGCTGGGATCTACTACTGTGCTGTTGTCTCATGTGGACACATACTGTTTGGAAACGGGACCAAGCTGGACCTCACAGGGAGTTTTCCTCCATTTGTGTATTTCCTGAGTGGAGCTCTGGCAGCTTCGCTGATCTTCCTCACATCGTACGCAATACACAAGATAAGGAACCACAAATGCAGGGCAAGAAATCCTCAGGACAGATCTGCTGTTGATTCAGGACCGGATGCAGAGGGGACAGAGGATCTCCATTACGCTGCTTTAAGACACAGTAAGCTCAACAAATCATGA

>NITR5b

ATGACTCTTCTGGTGTTTTCTGGCTGTGTGACATGTCTGCTCCTGGGAACAGTGGCTCTTTCTTGGGCTCAGAAACCGTCTGCATCTTTACAGTTTCAATCCGTTCTTGTTGGTGAGGAAGTGACTTTAAAATGCAATCGTCAAGGCACTGGGACAGATATAATTTATTGGTATAAACAACCTATAGGACTGAAACCACAACTCATGTCTGAGTATTTAGATTTTAGGAAAAATGGATCTTTTGTAGATGCTTTCAAGAACGATCCACGGCTTAAACTTGAAACAGACAAAGACCAACTCCACTTGAAGATCTCGAATTTGAAAATGTCAGACTCAGCCACCTACTACTGCATCAGTTCTGATTATTATGGGGTAAAACATCTGGAGGGCTACACTGTCCATGTGAAGGACTCTACTTCTGACATTCATGCTTCAGTGGATCAGTCGTCCTCTGAGAACTTCCATGCTGGACACTCTGTGACTCTGAACTGTACAGTACACACTGGGAGCTGTGATGGAGAACACAGAGTTTACTGGTTCAAAGACTCTGGAGACTCTCATCCAGGACTCATTTCCACTCATGGAGGCAGGAATGATCAGTGTGGGAGAAAGAACAACACACAAACACACAGCTGTGTCTACGAGCTGCACATGGAGAGGCTGACTGAGTCTCATGCTGGGATCTACTACTGTGCTGTTGTCTCATGTGGACACATACTGTTTGGAAACGGGACCAAGCTGGACCTCACAGGGAGTTTTCGTCCATTTGTGTATTTCCTGAGTGGAGCTCTGGCAGCTTCGCTGATCTTCCTCACATCGTACGCAATACATAAGATAAGGACCCACAAATGCAGGGCAAGAAATCCTCAGGACAGATCTGCTGTTGATTCAGGACCGGATGCAGAGGGAACAGAGGATCTCCATTATGCTGCTTTAAGACACAGTAAGGTCTACAAATCAAGAAGAGAAGGAAGATGCCGCCAATAA

>NITR5c

ATGACTCTTCTGGTGTTTTCTGGCTGTGTGACATGTCTGCTCTTGGGAACAGTGGCTCTTTCTTGGGCTCAGAAACCGTCTGCATCTTTACAGTTTCAATCTGTTCTTGTTGGTGAGGAAGTGACTTTAAAATGTAATCATCAAGGCACTGGGGCAGATATAATTTATTGGTATAAACAACCTCTAGGACTCAAACCACAACTCATGTCTGAGTATTTAGATTTTAGGAAAAATGGATATTTTATAGATGCTTTCAAGAACGATCCACGACTCAAACTGGAAACAGACAAAGACAAACACCACATAAAGATCTCAAATTTGAAAATGTCAGACTCCGCCACCTACTACTGCATCAGTTCTGATTTTTATGGGGTAAAACATCTGGGGGGCTATACTGTCCATGTGAAGGACTCTACTTCTGACATTCATGCTTCAGTGGATCAGTCGTCCTCTGAGAACATCCATGCTGGAGACTCTGTGACTCTGAACTGTACAGTACACACTGGGAGCTGTGATGGAGAACAGCGAGTTTACTGGTTCAAAGACTCTGCAGACTCTCATCCAGGACTCATTTCCACTCATGGAGGCAGGAATGATCAGTGTGGGAGAAAGAACAACACACAAACACACAGCTGTGTCTATGAGCTGCACATGGAGAGGCTGACTGAGTCTCATGCTGGGATCTACTACTGTGCTGTTGTCTCATGTGGACACATACTGTTTGGAAACGGGACCAAGCTGGACCTCACAGACTCTGCAGCTCCTCTGAACTCTGTGATTCTGAACACTTTTGTTGGATTATTGACCATCATGAGCGTCCTCGTCGCCTTTCTGCTGTGGAAGATTAAGAAGAGCAACGCCTGCACCTCCACAGAGGAAAGATCACCAGCTGCCCCCTTAAGAAGCACAGAGGCTGAGAGCGAAGATGCAGAAAACCTCCATTACGCTGCTTTAAATGTGAAAAAGTCAAAAAAAACAAGAAGACAGGAAAACGACTTAAACACAGTCTGTGTTTATGTGATTGTGAGACAACAGAACTGA

>NITR7a

ATGATTTCTAATCATTTTGTTGTTTTTCTGACTTGTTTGTCCTTCGGGGCCAAAGCTCAGAAGAATGTTCTAGAATCGTCCTCTGTAATCCAGAAAGGTGGTTTTCTATCTGTCTCCACTGGTGAAGCTGTTACTTTAGAATGTTCCTACAAAGGTTTAGATTTGACGTGGATCTTCTGGTACAAGCAAACACTGGGACAAAGACCTGAACCCATCTCTAGTTTCTACACATACGCTTCAGTGTTGACCTTTTTTGATCCATTCAACAACAATCCACGCTTCACTCTGGATACAAAAAATCAAAATCATCACTTGAGGATTTCAGATCTGCAACTTTCAGACTCGGCTACTTACTACTGTGCAGCACGACACGCAACCATCGTTACATTTACAGAGGGTGTTACAGTCAGGGTGAAAGGTCCAGGTGTAACCACGAAGACTTCAGTGGATCAGTCGTCCTCTGAGAACATCAATGCTGGAGACTCTGTGACTCTGAACTGTACAGTACACACTGGGAGCTGTGATGGAGAACACAGAGTTTACTGGTTCAAAGACTCTGCAGACTCTTATCCAGGACTCATTTACACTCATGGAGGCAGGAATGATCAGTGTGGGAGAAAGAACAACACACAAACACACAGCTGTGTCTACGAGCTGCACATGGAGAGGCTGACTGAGTCTCATGCTGGGATCTACTACTGTGCTGTTGTCTCATGTGGACACATACTGTTTGGAAACGGGACCAAGCTGGACCTCACAGACTCTGCAGCTCCTCTGAACTCTGTGATTCTGAACACTTTTGTTGGATTATTGACCATCATGAGCGTCCTCGTCGCCTTTCTGCTGTGGAAGATTAAGAAGAGCAACGCCTGCACCTCCACAGAGGAAAGATCACCAGCTGCCCCCTTAAGAATCATAGAGGCTGAGAGCAAAGATGCAGAAAACCTCCATTACGCTGCTTTAAATGTTAAAAAATCACAAAAATCAAGAAGACAGAAGAACGACTTAAACACAGTCTGTGTTTACGCGAGTGTGAAACAACAAAACTGA

>NITR8a

ATGATTCTGTTTTATGTAGTTTTTATTATCACAAATGGATATTGTGCGAATGAATATCATTTCACGACAAAGACGGTTCCTGTTGGAGAAGATGTTAAACTAACGTGCGCTCGCCAGACGAATGTTCTCTACAGAGAGAACTTATTTTGGATCAGGATTGTTTCTGGGAAAAAGCCTGAACTTCTGGGAGGAACAATGAACTTTGACTTTGACGATGAAATCAGGAAATCTCACATTACAGCAAAGCAAGAACCTGGGTCCTTTGTTCTGGAAATAAATGGAGCCATGGAAAGTGATGATGGTGTTTATTACTGCATTAAAGTTCAAAACCTGGATTTGACATTTTTGACTGGAACATTTCTGAGTGTCAAAGGCAGAGAACCACACATTGTTGCTGTCACTGAGAGGTTTTTATCTGATCAAGTCTATCCAGAAGACCCCATCACTCTAGAGTGTTCAGTCCTCTCCAGCTCTGACCATGAAACCTGTGCAGCAGAACAGAGAGTGTTCTGGTTCAAAACCCAATCAAACAAATCTCATCCTCATGTTATTTATGCTCATGGAAACAGTTCTGATGAATGTTTGAGGACTCCTGAAGCTCCCTCTGTGCAGAAATGTGTCTACAGCTTTAACAAGAATTTCATCTCCTCTGATGCTGGGACTTACTACTGTGCTGTGGCTGCATGTGGAGAGATCTTTTATGGAAATGGAACAACACTGACCGAACCCCAGATGTGGGATTTACAAACAGCCAACACTGTTCTCCTTGTCTTGTTTGCCACTTTCTCTGCCAGCATATTTGTTATAATTTTCTTACTTTATAAAATAAAAAAGAAGTCAGGCAGTTCTTGCAACGATATCTTACACTCCTTAGATGACCAAAATCATCAAAAGAGTAAAAATGATTCACTGACATATTCAGCTGCAACCTTCACGAAGAGGGAAGCTGGTAGATTTGCAACATCCCAGGAGGCCCTTTAA

>NITR8b

ATGATTCTGTTTTATGTACTTTTCATTATTACAAATGGATATTGTGCGAATGAATATCATTTCACGACAAAGACGGTTCCTGTTGGAGAAGATGTTAAACTAACGTGCGCTCGCCAGACGGATGTTCTCTACAGAGAGAACTTATTTTGGATCAGGATTGTTTCTGGGAAAAAGCCTGAACTTCTGGGAGGAACAATGAACTTTGACTTTGACGATGAAATCAGGAAATCTCACATTACAGCAAAGCAAGAACCTGGGTCCTTTGTTCTGGAAATAAATGGAGCCATGGAAAGTGATGATGGTGTTTATTACTGCATTAAAGTTCAAAACCTGGATTTGACATTTTTGACTGGAACATTTCTGAGTGTCAAAGGCAGAGAACCACACATTGTTGCTGTCACTGAGAGGTTTTTATCTGATCAAGTCTATCCAGAAGACCCCATCACTCTAGAGTGTTCAGTCCTCTCCAGCTCTGACCATGAAACCTGTGCAGCAGAACAGAGAGTGTTCTGGTTCAAAACCCAATCAAACAAATCTCATCCTCATGTTATTTATGCTCATGGAAACAGTTCTGATGAATGTTTGAGGACTCCTGAAGCTCCCTCTGTGCAGAAATGTGTCTACAGCTTTAACAAGAATTTCATCTCCTCTGATGCTGGGACTTACTACTGTGCTGTGGCTGCATGTGGAGAGATTTTTTATGGAAATGGAACAACATTGACCGAACTCCAGATGTGGGATTTACAAAAAGCCAACACTGTTCTACTTGTCTTGTTTGCAACATTGTCTGCCAGCATCTTTGTCATAATTTTCTTAATTTATAAAATGAAGAGAAAAGCAGGCACTACTTCCAATGATGGCTTACACTCCTCTGATGACCGGAAACACGAAAAGAATAAAGAAGATTCACTGACATATTCAACACCAACCTTCACTCAAAGGAAAGCTGGCAAAGCGAAAAGAAAGCAGAAAACACCTCAGGAGACCTTTTATTCTGATATCAAAAATCTGGGATAG

>NITR9a

ATGCAGCTGCGTGTGATCCTCTGTGGATTATTCCATCTGTCAGCTGTGATCTGGGCTGGAGCAGTTAAACAGGACACTGGTGTCAGATCCGTCTCTGTCGGGGAAAATGTGACTCTGCAATGTTTCTATGAAAACGTGATGGCGATGCACTTCTCCTGGTACCAGCAGCCTCTTGGAGGAAGACCTGAACTCCTGTCTTTCTTTTACAAATACGATAACCCATCTAAAGTGGACCACTGGTTGCAGAAGAAACCCCGATTCTCTCTGCAGAGGGAGGAAGGCATCAACCACTTACACATCTCTGATGTGCAGCTTTCAGATTCAGCCACTTATTTCTGTGGAAGCTCGCACTCCAACATGGTGGAATTTGGGGATGGCCTCTTTCTGAGCGTTGAAGAAAAAAGCCCCACAGAAATCATCATCCAGGAACCGACGTCAGAGACCATCCAGCCTGGAGGCTCCATCACCTTCAGCTGTACCGTTCATTCAGGGAACTGTGGGGAAGCACAAACTGTCTGCTGGTTCAGACGTGGCTCTCAACCGGGGGTCCTCCACACACAGAGAAAAGACTGCAGACCTGTCGCTGCTCCAGGGCCTCCTTCACAGAGCTGCACCTACAGTTTGCAGAAGAAAGACCTGAACTCCTCTGATGCTGGAACCTACTTCTGTGCTGTGGCCTCTTGTGGGAAAATGCTGTTTGGCAGTGGAACCAAGCTGATCATGACAAATCAAACTGAAGGCCAAGCAGCTCAGATTAAAGTGTTAGTCCAGCTCTCTGTCATCAGAACCGGGGTTCTTCTGCTCTTTCTCCTCAGTTGTGTGCTCTTTGTCAGAAAAAGTGATCCAAGTCCATGA

>NITR9b

ATGCAGCTGCGTGTGATCCTCTGTGGATTATTCCATCTGTCCGCTGTGATCTGGGCTGGAGCAGTTAAACAGGACACTGGTGTCAGATCCGTCTCTGTCGGGGAAAATGTGACTCTGCAATGCTTCTATGAAAACGTGATGGCGATGCACTTCTCCTGGTACCAGCAGCCTCTTGGAGGAAGACCTGAGCTCCTGTCTTTCTTTTACAAATACGATGACCCATCTAAAGTGGACCACTGGTTGCAGAAGAAACCCCGATTCTCTCTGCAGAGGGAGGAAGGCATCAACCACTTACACATCTCTGATGTGCAGCTTTCAGATTCAGCCACTTATTTCTGTGGAAGCTCGCACTCCAACATGGTGGAATTTGGGGATGGCCTCTTTCTGAGCGTTGAAGAAAAAAGCCCCACAGAAATCATCATCCAGGAACCGACGTCAGAGACCATCCAGCCTGGAGGCTCCATCACCTTCAGCTGTACCGTTCATTCAGGGAACTGTGGGGAAGCACAAACTGTCTGCTGGTTCAGACGTGGCTCTCAACCGGGGGTCCTCCACACACAGAGAAAAGACTGCAGACCTGTCGCTGCTCCAGGGCCTCCTTCACAGAGCTGCACCTACAGTTTGCAGAAGAAAGACCTGAACTCCTCTGATGCTGGAACCTACTTCTGTGCTGTGGCCTCTTGTGGGAAAATGCTGCTTGGCAGTGGAACCAAGCTGATCATGACAAATCAAACTGAAGGCCAAGCAGCTCAGATTAAAGTGTTAGTCCAGCTGTCTGTCATCAGAACCGGGGTTCTTCTGCTCTTTATCCTCAGTTGTGTGCTCTTTGTAAGAAAAAGTGATGCAAGTCCATGA

>NITR9c

ATGCAGCTGCGTGTGATCCTCTGTGGATTATTCCATCTGTCCGCTGTGATCTGGGCTGGAGCAGTTAAACAGGACACTGGTGTCAGATCCGTCTCTGTCGGGGAAAATGTGACTCTGCAATGCTTCTATGAAAACGTGATGGCGATGCACTTCTCCTGGTACCAGCAGCCTCTTGGAGGAAGACCTGAGCTCCTGTCTTTCTTTTACAAATACGATGACCCATCTAAAGTGGACCACTGGTTGCAGAAGAAACCCCGATTCTCTCTGCAGAGGGAGGAAGGCATCAACCACTTACACATCTCTGATGTGCAGCTTTCAGATTCAGCCACTTATTTCTGTGGAAGCTCGCACTCCAACATGGTGGAATTTGGGGATGGCCTCTTTCTGAGCGTTGAAGAAAAAAGCCCCACAGAAATCATCATCCAGGAACCGACGTCAGAGACCATCCAGCCTGGAGGCTCCATCACCTTCAGCTGTACCGTTCATTCAGGGAACTGTGGGGAAGCACAAACTGTCTGCTGGTTCAGACGTGGCTCTCAACCGGGGGTCCTCCACACACAGAGAAAAGACTGCAGACCTGTCGCTGCTCCAGGGCCTCCTTCACAGAGCTGCACCTACAGTTTGCAGAAGAAAGACCTGAACTCCTCTGATGCTGGAACCTACTTCTGTGCTGTGGCCTCTTGTGGGAAAATGCTGCTTGGCAGTGGAACCAAGCTGATCATGACAAATCAAACTGAAGGCCAAGCAGCTCAGATTAAAGTGTTAGTCCAGCTGTCTGTCATCAGAACCGGGGTTCTTCTGCTCTTTATCCTCAGTTGTGTGCTCTTTGTAAGAAAAAGTGATGCAAGTCCATGA

>NITR10b

ATGCATGTTGTACTTTGTTCACTGCTGATACTTCAACTTGGATGTTGTGCAGATGAGGGATTTGAAACAAAGACTGTAGACTCTGGAGAAGATGTGACCCTGCTCTGTAACAACTCACGGCTGAACTTTGGATTCTTTTTCTGGATGAAAAGTGTTCCCGGACAAATGCCTGAAATCGTAGGAAAAAGATTCGGCAAAAGTGACGAATTCAACAAGATTCATCACTTTACAACCAAAGAAGAAGACGGAAGATTCTTTCTACAAATTTCAGAAGCAAAGCCGAGTGACTCAGCCTTCTATTACTGTTTTACATTTAAAAACTACAAAATAACCTTTATGAAGGCAGTGCTTCTTAGAATTAAAGGACCGCGGTCTGATTTTCCTGCAGTGGTTCAAAGTTCTGTTGATCTGGATTCTGCAGGAGACGTTGTGGCTCTGCAGTGTTCAGTTCTCTCTGAGTTTAAAAATGATGCATGTCCAGAGGAACAGAGAGTGTTCTGGTTCAGAAAGACTGAAGGGGAATCTCATCCCACTTATATTTACGCCAGAAGAAGCAGTGATGGTGACTGTGACGGGGGTACAGAGACTCAGCCTCTGCAGAGTTGTGTCTACAGCTTCTTGAAGAACGTCAGCTCCTCTGATGGCGGACTTTACTACTGTGCTGTGGCTGCATGTGGAAAAGTGGTTTTTGGAAATGGAACAAAACTGGATGTTCAAGTTGATGGCACATATGACTCCAGGAACAACAATAGATATTTCTCTCTGTTTGTTGGAACCTTGGTTTTGAGTCTCACAATGGTAACATTTTTGATTTGGGTTGTATCAAAAAAATCCTGTGATGTCTGCAAAGTGTATTTGGCCTGTAAAGCAACATCTGACACAGTCAATGATGAACAGCAAAGCCAGAAGATAAATCAAAACAATGTGGTTTATGCTGCAACAGTCTTTGCCAAGAAGAAAGCTGATAAGACCAAGGGGAAGAAAAGGAGCAATAAAGGAAGAGACCATCTACAGTGA

>NITR11a

ATGAGGAGCCTCATCGTAGTAACAACTTTTCTTCTCTGCAGCCGCAGCTGCTTCTGTGAGTCTAAGACTGTGGAGGTCCAGTCTGGAGAAAACGTCAGTCTGCTCTGCTCTGATTTCACCAAAAACAGACAACAGACTGACTGGTTCAGAGTGGTCAAAAGCTCCAAAGTCAGCTGTATCTCCTCCATGTTTGGTGTTGATGGTGATCCTTCCTTCTGTGATGGATTTGATGGTGGAAAATTTGAAATGAATTCCAACAGCAGCTCTGTGTCTCTGAAAATCAGTGGAGTGGACGAGTCTGACTCTGGGCTGTACTTCTGTGGATTCTACAGAAACAGACATACAGTCATTGGAGATGTAACACAACTGATCATTAAAGACTCTACAGTTTTCATGAAACATCCATATACTAGCCTTCTGACTGTGATTCTGGCTGTTCTGATGTCGTTTTTCATTTTTGGTGCTCTTGGTTTGGTTGTTATTTTTGAACATCATTGTGCAGCAAAGGAAAATCTGCACACGGACAGCCTGAAGACTCAAGACTCTTCTGACCCAAACTCTGCTGCTCTTAAGTTTTCAAAATCCAGAAGAAGTGGACGACCTGCAGAAAGCAGACAGTTGGAGACTTGTGTGATGTATTCTGTCAGAAGACAAACTTACAGTTCTGTTTGA

>NITR11b

ATGTGGAGCCTCGCCATAATAACAGTTTTCCTGCTCTGCAGCCGCAGCTGCTTCTGTGAGTCTAAGACTGTGGAGGTCCAGTCTGGAGAAAACGTCACTCTGCTCTGCTCTGATTTCACCAAAAACAGACAACAGACTGACTGGTTCAGAATGGTCAACAGCTCCAAAGTCAGCTGTATCTCCTCCATGTTTGGTGTTGATGGTGATCCTTCCTTCTGTGATGGATTTGATGGTGGAAAATTTAATATGAGTTCCAACAGCAGCTCTGTGTCTCTGAAAATCAGTGGAGTGGATGAGTCTGATTCTGGGCTGTACTTCTGTGGATTCTACAGAAACAGACATACAGTCATTGGAGATGTAACACAACTGATCATTAAAGAGAGGCATGTGAGGATGGACCTGCTGAGTGTCCTCCTGGCTGCTCTGACTGTCCTCCTCTCTGCAGTGGTTGTTGTTCTGGCCGTAAAGATCAGGAAACTTCTGACAGCTGCACGAAAAGAACAAGAAAATACAAAGAATCTGGACTCGAATGATCTGAACTACGCCGCTCTCAGTTTCAATCAGAAACCAAAGAAAGGCGGCAGGTCTCCATCAGACAGAGAGCTGCAGCCACATGTTCTGTATGCTCCCACCAGGTAG

>NITR12a

ATGGGCTTGACCTCAATACTGTCTTTTCTCTGCAGCTTCAGTTTGATCTGTGTGTCACTGTCTGAGTTTGTCGTCGTAGAGGTTCAGCTTGGAGGAGAAGTCTCACTGCTGTGCTCCAACCTCAGTAACATCATGTCCAACATTTTCTGGTTCAAGTCGGCCAAAAGATCCAACACCACCCGCATCGCATCTATGCCCACTGCTGAGTCCAATGCTACAGTTCTGGAAGATTTTAAAAATGGAAGATTCCACATGTCCTCCAACACCACCCATGTTTTCCTGAACATCAAGGAGTTTAATATCTCTGACTCTGGACTGTATTTCTGTGGATTGAACACAATTAATTACAATTTTGATGCAACATTTCTGCAAGTAGAAGAAACACCTGCCTTTAATCTGGTGGTTGGAATCCTTGGAAGTGTAATTTTTGTTCTTGTGATGGTCATCATCTTTCTGCCGGTCAAAATCAAATCTTTTCAGAAAGCTCCGTCTGAGCGCCAGCACAGAGAGAGCCTGGAATCCAACGTTCGATACTATGCAGCCCTGACATTCCAGCCAAAACCTGAAACCCAAAGCATATCTGTATATAAAACCGATTCAGATCTGTGTTTTTGGTCTTAG

>NITR12b

ATGGGCTTGACCTCAATACTGTCTTTTCTCTGCAGCTTCAGTTTGATCTGTGTGTCACTGTCTGAGTTTGTCGTCGTAGAGGTTCAGCTTGGAGGAGAAGTCTCACTGCTGTGCTCCAACCTCAGCAACATCATCTCCAACATTTTCTGGTTCAAGTCGGCCAAAAGATCCAACACCACCCGCATCGCATCTATGCCCACTGCTGAGTCCAATGCTACAGTTCTGGAAGATTTAAAAAATGGAAGATTCCACATGTCCTCCAACACCACCCATGTTTTCCTGAACATCAAGGAGTTTAATATCTCTGACTCTGGACTGTATTTCTGTGGATTGAACACAATTAATTACAATTTTGATGCAACATTTTTGCAAGTAGAAGAAACACCTGCCTTTAATCTGGTGGTTGGAATCCTTGGAAGTGTAATTTTTGTTCTTGTGATGGTCATCATCTTTCTGCTGGTCAAAATCAAATCTTTTCAGAAAGCTCTGCCTGAGCGCCAGCACAGAGAGAGCCTGGAATCCAACGTTCGATACTATGCAGCCCTGACATTCCAGCCAAAACCTGAAATCCAAAGCATATCTGTATATAAAACCGATTCAGATCTGTGTTTTTGGTCTTAG

>NITR13

ATGATTTCTAAGCTCTTTGTTTTCTTTCTAACATGTGTGTTTGTAGGAGCAGATGGTCAGAAGACTTATCTGAAATCTTCATCCTTTGTGTGGCAGAAAAGTGGTTTTCTATCAGTCAACGTTGGTGACACAGTTACTTTAGAATGTTCTTATGAAGGTTATGAATTAACATGGATTTCCTGGTACAAACAAAGTTTGGGAGGAAAGCCTGAACTTATGTCCAGTTTCTATGCGTACAGCACGGAAGTGACCTTTTTTGATGAATTCAAGGACAATTCACGCTTCAAACTGGATATAGAAAACAAAAATCATCACTTGAAGATTTTGAATTTGCAATTTTCAGACTCTGCTACTTACTACTGTGCAGTGAGCTATGCAATGATCCTGAAATTCACTGAGGGTGTTACAGTCAATGTAAAAGGTTCAGGCTTAACTGTCATGAGTTCAGTGGATCAGTCGTCCTCTGAGAACATCCATGCAGGAGACTCTGTGACTCTGAACTGTACAGTACACACTGGGAGCTGTGATGAAGAACACAGAGTTTACTGGTTCAAAGACTCTGAAGACTCTCATCCAGGACTCATTTACACTCATGGAGGCAGGAATGATCAGTGTGATAGAAAGAACAACACACAAACACACAGCTGTGTCTATGAGCTGCCTATAAAGAAAGCGACAGAGTCTCATGCTGGGATCTACTACTGTGCTGTTGTCTCATGTGGACACATACTGTTTGGAAACGGGACCAAGCTGGACCTCACAGGTGATGATGATTCTGATAGTTTCCATACTCCCTTCCTGGTTTACCTTTTTACATCACTGATAGTTATCATCTGTCTTCTTGTCTTGGTGATTTTTCTGTTGTGCAAGATGAGACAAAGCAACAAATATCCATCCACAGAGGAGAGATCGGCAACTCTGTCCTTAACAAGCACAAAGGTTAAAGTTCAAGAAGCAGAAAGCCTCCAGTATGCCACTGTAGATGTGAAGCGGATGAATCGATCAAAAAGACAAAAGAACGACACCAGCAGCCAGTGTATTTACACAAGTGTGAGGGAGAAGAGCTAA

>NITR14

ATGGAGACATCTGCTCACATTGTTTTCTTTTTGACACTTTTGCTTTCAGTTCAAATGACTCTTCTCGCATCTTCCTCATCTGTGCATGCAGACAGTGAATTTGTATCCGGTCTTATTGGTCAAACTGTGACTTTACCATGTGACTACAACAGGAGTGATTTCATTTGGGTTGCATGGTACAAACAAATGGTGGGAAAGAAGCTGGACCTCATTTCACAAATGTTTAGGTATCAAAAAACAGTCATGAAGAATCCACGTTACAGTGTGAAAAGTCAAAACCAAAAATATCACTTAAATATTTTGAACCTACAAGACTCTGACTCCGCTACGTACCACTGTACCATTTGTTTTGCTGCATCAATAGATTTCTTAAATGTGGTTACAGTCAATGTAAAAGGTTCAGGTTCAACCATCCAGACTTCAGTGCATCAGTCGTCCTCTGAGAACATCCATGCAGGAGACTCTGTGACTCTGAACTGTACAGTACACACTGGGAGCTGTGATGAAGAACACAGAGTTTACTGGTTCAAAGACTCTGAAGACTCTCATCCAGGACTCATTTACACTCATGGAGGCAGGAATGATCAGTGTGAGAGAAAGAACAACACACAAACACACAGCTGTGTCTATGAGCTGTACATGAAGAACCTGACAGAGTCTCATGCTCGGATCTACTACTGTGCTGTTGTCTCATGTGGACACATACTGTTTGGAAACGGGACCAAACTGGAACTCACAGACTCTTCAGCTCCTCTTAACTCTGTGATTCTGAACAGTTTGGTTGGATTATTGACGGTCATGAGCGTCCTCGTTGCTTTTCTGTTTTTTCTGCTGTGGAAAAACCACAAGAGCAACAACTGCACCTCCACAGAGGAAAGATCGCCTGCTGCCCCCTTAAGAAGCTCAGAGGCTGAGAACAAAGACGCAGAAAGCCTCCATTATGCTGCTGTTAATGTTAAGAAGTCCAGCAGAACAAAAAGTCAGAAGAACGACTCCAACACCGACTGTCTTTATGCGAGTGTGAGACAGCAGAACTGA

>NITR15

ATGAAGACATCTGCTCAGTTTGTCTTATTCCTGACGGTGTTGCTCTCAGTTGGAAAGATTCTTCTGACATCGTCCTCGTTGTTGCATCAACAGACTGATTTCAAATCAGCTCACAAAGGTGAAACTGTGTTTTTACCATGTATCTATGAAGTGGATGATCTAATATGGGTCTCGTGGTACAAACAAGCTTTGGGACAGAAACCAAAGCTGGTCTCAAGATTTTTCAGATATAGAAAACAACCCAATTTCTTCCATGACTTTAATGACAATCCACGCTTCTTACTGCATACAGAAAACCAAAATAATCACCTGACAATTTTAGATCTACAATATTCAGATTCTGCTACTTACCTATGTGTAGCAAACTTTGGAAAAGATCTGAATTTCTCATCAGCTTTTACCATCGATGTAAAAGGTTCAGGTTTAACCATCCAGACTTCAGTGAGTCCGTCGTCCTCTGAGAACATCCATGCAGGAGACTCTGTGATTCTGAACTGTACAGTCCACACTGGGAGCTGTGATGAAGAACACAGAGTTTACTGGTTCAAAGACTCTGAAGACTCTCATCCAGGACTCATTTACACTCATGGAGGCAGGAATGATCAGTGTGAGAGAAAGAACACACAAACACACAGTTGTGTCTATGAGCTGCACATGAAGAACCTGACAGAGTCTCATGCTGGGATCTACTACTGTGCTGTTGTCTCATGTGGACACATACTGTTTGGAAACGGGACCAAGCTGGATCTCATAGACTCTGCAGCTCCTCTGAACTCTGTGATTCTGAACACTTTGGTTGGATTATTGACGGTCATGAGCGTCCTCGTTGTTTTTCTGCTTTTTCTGCTTTGGAAAATCAACAAGAGCAACAACTGCACCTCCACAGAGGAAAGATCGCCTGCTGCCCCCTTAAGAAGCACAGAGGCTGAGAACAAAGACACAGAAAGCCTCCATTATGCTGCTGTTAATGTTAAGAAGTCCAACAGATCAAGAAGTCAGAAGAACGACATAAACACAGACTGTGTTTATGCCAGTGTGAGACAGCAGAACTGA

>NITR16

ATGGTGACACCTGCTCAGTTTGTCGTCATTCTGACATGTTTGCTTTCAGGGAACACTGCACAGAGCATTTCTCTGGGATCATCTTCATCTGTTCTTCAAGAAAGTGTTTTTAAATCAGCTCACATTGGAGAAACTGTGACTTTACCATGTTTCTATAAAGAATTTTATTTAAAATATGTCTCATGGTACATATATATTCTGGGGAAAAAACCAAAACAGTTATCTTATTTACGTAAATATGACAAAAATGTAACTCTTAGCGATGAATTCAAGAACAGGTTTACTTTAAAACCAGGAAACCAAAGTAGTCATTTGATAATTTCAGACCTGAAACAATTGGACTCAGCTACTTACTTCTGTATAGCTGGTTATCAAACACACCTGACTTTCACAGCAGCTTTTACAGTTGATGTAAAAGGTTCAGGTTTAACCATCCAGACTTCAGTGGATCAGTCGTCCTCTGAGAACATCCATGCAGGAGACTCTGTGACTCTGAACTGTACAGTACACACTGGGAGCTGTGATGAAGAACACAGAGTTTACTGGTTCAAAGACTCTGAAGACTCTCATCCAGGACTCATTTACACTCATGGAGGCAGGAAAGATCAGTGTGAGAGAAAGAACACAAAAACACACAGCTGTTTCTACAAGCTGTACACGAAGAACCTGAATCGAACTCATGCTGGGATCTACTACTGTGCTGTTGTCTCATGTCAGCCGAACTCTTTGTTGGTGTATTTCTTAACTGGATCTTTGACCTTCATGAGCATCGTTGTTATTTTTCTTGTTTACAAGATTACAAAAGATATTTGCTGCCAGTCTAAAGATCAAATATCAGCAGCTGGCTCTACACCACAAACAGAGATTGAGAGCGAAGACACAGAAAGTCTTCATTACGCTGCTATCAATGTTAAGAGGTCCAACAAATCAAGAAGACAGAACGGCTCCATCACAGACTGTATTTATGCGAGTGTGACACAGCAGAACTGA

>NITR17

ATGGCATCCATACCATGTGCGATCTTTCTGACAGTTTTGTTCTCTGGTGAAATGGCTCAGACAAAAACCTCCTCCCTGTCATCATCTCTCCGTCATGACACAGATTTTTTGACCGTTAAACCTGGAGACAACTTGAGTTTGAAATGTTTCTATGAAGATGTGGTTGATGCTCGATTTTATTGGTATAAACAAAGCTTAGGGCAGAAGCCGAAGCTCATTTCTATCTCGTACAAATATGAAAACAAAGGAGTGTTCCATGGGGAATTCCGAAACAGTTCACGCTTTACACTGGATAATAGAAAAGGGAACAATCACCTGATGATTGCAAACCTACATTATTCAGATTCAGCAACGTATTACTGTGCAAGCAGCTATTTGTACAACTTTGAATTCTCAGATGGTATTACAGTCAATGTAAAAGGTTCAGATTTAACCATCCAGACTTCAGTGGATCAGTCGTCCTCTGAGAACATCCATGCAGGAGACTCTGTGACTCTGAACTGTACAGTCCACACTGGGAGCTGTGATGAAGAACACAGAGTTTACTGGTTCAAAGACTCTGAAGACTCTCATCCAGGACTCATTTACACTCATGGAGGCAGGAATGATCAGTGTGAGAGAAAGAACAACACACAAACACACAGCTGTGTCTATGAGCTGCCGGACCTGACAGAGTCTCATGCTGGGATCTACTACTGTGCTGTTGTCTCATGTGGACACATACTGTTTGGAAACGGAACCAAGGTGGACCTCACAGCTGAGGTCCCGTATCAAAACTATCTCTGGAGTGGTGCGCTCTTTTTCACGACTCTTCTGTGTGTTTTCCTGGGGATCTCACTTTGCTTGATGACTAGATCACACAACAGAAAAATGTCAGGGTCATCACAACCCCCCAAAGGTGCCATGGGTTTCAAGTCAACAGAAAAAGTCTACCATGCAGCCGTCTGTACCAACTTGGCAAACAGGTCCAGACGACTAAGGGATCCCACATGGAGCGAATGCGTGTACAACAGCATGAAGCAGTGA

>NITR18

ATGATCAGAGGACTGGCTGCTTTGATTTTGCTCAATTTACTAACTGTGGTTCAAAACCTGGAGCTTCTTCAGCAGATCTCAGCTGAACTTGGGGACAATGTGACTCTGACTTGCTTAACTTCTGGTGTTGATCATGGTTTATTCTTTTGGTACAAGTTTCAGTTTGGGTACAGGTTCCAAATGGTCAGTTCAGGCAATTTTGGTCAACTAAAACTGGAGCAACAATTTGATACCCCAAGATTCAATATGGTGAATGTAGGTAATATATATTCCCTCAACATAAGAAATATTAGCAAAGAGGATGAAGGCATGTACATCTGTCAGGCAGGAGCAGCCTACAAACTCCGCTTCATCAGTGGCAATCGTTTGATGGTAAAAGATCCAAAAAAGAGACCTAAAACCATATTTGTAAACCAAAGTTCCATTATGGAGGCAGTTCTTTCAGGCCAGTCAGTAAATCTCTTCTGTTCAGTTTTGTTAAACACCAGAGAAAACTCAGACCTGTGCTCAGGTAAACACAGAGTTTACTGGTACAGAGCTGGATCAGAATCACATCCCCACCTCATTTACACAACCAGATCCAGCTGTGATGTTCAAAAAGGAATGAGATGTATCTACAATCTAACCCAAACAATAAGGGGCCTGTCGGATTCTGGTGTTTACTACTGTGCTGTGGTCTTGTGTGGAGAAATCCTGTTCGGTGAAGGAACCAAAGTGCAGATAAAACAACTGTCCCCTGATGCCATCGTCCTTGGATCCTTGTTAGCCTGTTGTGTACTCATGAACATCACTCTAATTGTGATGAGAACAAAGCAAAAGTGTGAGCAGTGCAAAGATAGTTATTATATATTGGTCATGCTATTCTACTTTTGGGTCACCTGTACAAATGATGGTGAAGAAGATGGACAAAGTGGACAAAGAGCAATAGCATCACAACACAACTGTTTGGATTTAAACAAGAGAAACAATCTGAAAAGTAAATTACCGGGAACTCTAACGACACACCCTTTTATTAATCAGATGAAGAAATGA

>NITR19

ATGAGTGTAAGACTGGCTGCCTGCCTTTTTCTATGTGTAGTTGCTGTGCCACAGACCCTGCAGCTTTCTCAAAAGATCTCCATTACTGAGGCGACCTTTGGTGAGAATGTGATCATGAAATGTACAACTGTTGGGTTGGAACAGCGAATGGTTTACTGGCATAAACTGCAGTTTGGTTTTATGATCCAAACTATTGCTTTTGGCAGTAGTCCAGACCTTCCTCTTAAGGAAGGGTTCAACAACTCTAGATACAGTGCCAAAAAAGAAGGAAATGAGTATTCCCTCGAAATAAGAAACGTCAGCAAAGAGGATCAAGGAACTTACTTCTGTCAGGCAGGAACATCATACACAATGACTTTCATTTATGGCAGTCATTTGGTCCTAAAAGGATCCAATCTGGAATCTGTGTCGCCGGGAAGCAAAGTGAATCGGCAGTGTTCTCTTCTTTGGCAGCCCGAGAAAAACCCAGATCGGTGTCTGGGAGAACAGAGAGCTTACCTTTACAGAGCAGGATTTGAGTCTGAGCCAGACATCATTTATACAACCAGTTCCCGCTGTGATGATCAAGAAGACAAGAGCTGTGTCTACCATGTGTCCAAACCTATGAAGAACTCTTTAGATCCTGGCCTCTACTCCTGTGCTGTGTCTTCCTGCAGAGAGATCCTGTTTGGTGAAGATACTGAAGAGCAGATAAGGCAAGGCGTGTGTCCGTATGTTCTCATACTCGGGACACTGCTGGCCTGCTGTGTCCTCGCAAATATCATTTTAATTGTGACCTGGAAAAAGCAAAATCCAGTTTGTGACAAGTGCAAAGATGGTAAAGCTCATACTAATGCCGAAAATGATGGACCAGCTGGGGATCAACAGGCTGACTTGGGTGAGGAAGATGACGGATTGCATTATGTCGCACTTGAGTTCTCCTCGAGGAAGTCAAAAAGACAGAGAAGCATGAGGAGGGAGTCCAAGGAGACCTGTATTTATGCAAACAAAAGAGATTATTAA

>NITR20

ATGATCAGAGGGCTGGCTACTTTCATTCTTCTAGGGGGAGTATTTGTGATTGAAACCCTGGAGCTTCCTGAAGAAATCCCCTTGATTGAGGCAGAACTTGGTGATAATGTGACTTTCACATGTTCCTCTCAGGATTTTGACCAGGCACTGATGTACTGGTATAAGTTTCAATATGGTTATGTGATCCATACAATTTTAAAGAGCAGTTTTGGGAAATTTAATGAACATTTTGACGACTCAAAAATCTATACTGTAAAAAAGAATGATGATAAATTAATTCTCACAATAAAAAATGTCAGCAAAGAAGATGAAGCCGCTTACTTTTGTCAGGCAGGTTCTTCATACAACATGGAATTCATCAATGGGATTCATTTATTTGTGAAAGGTCCTAAAAATGAACTGAAATCTGGATCTGTGAAACCGAGTCCAAAACTGGAGTTGGTCCTGCTGGGAAACACAGTGAATCTGCAGTGTTCAGTTCTGTCTGAAAAGAATCTGTGTGCCAGAGAACGCAGAGTTTACTGGTACAAAGCTGGATCTAAATCTCCCGCAGACATCTTTCATGCAACCAGTCCCAGCTGTGATGATCGAGAAGGGAGATGTGTCTACAATCTGTCCAAAACCATACAGACTTTCTCCGATTGTGGCGTTTACACCTGTGCTGTGGTCTCATGTGGAGAAATCATGTTTGGTGAAGGAACCAAAGTGCAGATGGCACAAGACATGTTGTGTACCAATGCTAAACAACATATCAGCATATTTGGGACACTGTTAGCCTGCAGCATACTTGCAAATATTGCATTATTTCTTGCAAGGAAGAGGCAAAAAGGAGTTTTCGGGCTCTGCAAAGGCGATGCTGCAAGGTCCAGCCAGGCTGAACAGCTTAGATCAGCTGAAGACCAACCAAACCATGGGGGTTTTGGGGAAACTGAGATGTGTTATGCAGCCTTGGATTTCACTCCAGGACAATTAAGAAGACCAAAAATCACAGAAAACCCAGCAGAGGACTATATATACTCACAGACAATGTTTAAAAGAAGAGTTCGTGAAGAGTAA

>NITR21

ATGATCGGAGAGCTGTCTGCTTTCATTCTTCTCACTGCAATATTCGTGACCCAGCCGATGGAGACTCCTTCAAAGATCTCTTTGACGAAGGCTGAACTGGGCGATGATGTGACTCTGATTTGTTCAACTACTGGCATTGATAATAACCTACTCAGCTGGTTTAAGTATGAGTTAGGATATGTGATTAAAACTGTTGGTAAGATTAATTATGGAATAGTGCAAATGTATGGGCAGTTTAACTCTTCAAGATTCGGGATCATTCCAGAAGCTAAAGGGGGTTCTCTCAGCATAAGTAATGTCAGCCAAGAGGATGAAGGAACATACTTATGTCAAGCAGGATCATATTTAACAATGAGATTTTTTAATGGCAGTCACCTGGTTGTAAAAGATCCCACAAAGAAACAAAATGCCGTTCATGTGCAACAAAGTTCAGATGCAGAGTCAGTTTTCCAGGGAAACACATTCAATCTGCAATGTTCAGTTCAGTCAAAGATCAAGAAGAACCCAGATCCGTGTTTGGGAGAACACAGAGTCTACTGGTACCAGGCTGGATCAGAAACTCATGCAGACATCATTCACGCAACCAGTCCCAGCTGTGATGACCATGGAAGAAGATGCGTCTACAATCTGTCCAAAACCCTACAGAACTTCTCCGATTCTGGCATTTCCTCCTGCGCCGTGCTCTCATGTGGACAAATTCTGTTTGGAGAAGGAAACAAAGTGCGGATGAAACAACCTGAGTTCCTTTATCTCCTCATACTTGGAACACTGTTGGTTTGCAGCGTCCTTGTGAATACCGCTTTAATCGTCATCGTAGGAAAGGGTTGTTGTGAGCACAGCAAAGATGCTGCAGCTCCGAGGCATCGTAAACGAGAGAAATCAGCCGAGGATGATACCAACCTGGATGATGAAGAGGCTGAGCTAAACTACGCAGCATTGAGGTTTCCACCGAAAACAAAAAACCTGAGGAGAACAAATGAGTTTACAGAAGACAATTTCTAG

>NITR22

ATGCAAGGAGGACAGATCACTTTCATTCTTCTCTGTGCATTATCAGTCACTCAATCCCTTGCACCGGAACTGGTCCACATGATTGAGGCTGAAACTGGCAGTAATGTGACTTTGAAATGCTCAGCCTCTGGAACTGACCAACAGTTGTTCTTTTGGCATAAACTTCAGTTTGGGTATATGATTCAAAGTGTGGCAACAGGCAACATGGCAAACATAGCACTAAAAAAAAATGTTGAACGCTCAAGATTTAATGTCACAAAAGTGGGGGATGTGTATTCTCTTTTTATAAGAAATGTCAGCAAAGAAGATGAAGCAATGTACCTTTGTCAGGCAGGAGCATCCTACGCAGCAAAATTCACTAACAGCAGTCAGCTAGTTGTAAAAGGTCCCAAAAAGAAACAAAAATTTGTTAAACAGAGTCCCGACGTGGAGCTGGTTCTGCTGGGAAACACAGTGAACCTGCAGTGTTCGGTCCTGTTAAACACCAGAGAAAGCTCAGATGAGTGTTCAGATGACCACAGAGTTTTCTGGTACAAAGCTGGATCAGAAAAACATGCAGACAATATCTATATAACCAATTCAGGCTGTGATGTTCAAAAAGAAAGGAGATGTGTCTACAATCTGCCCAAAGTCGTGAAGAATTCTTCGGATTCCGGCGTTTACTATTGTGCTGTGCTCTCACGTGGAGAAATCCTGTTTGGTAACGGAACTGAAGTCCTGATAAAAAAAGAACTCTGCCCGTATGTCATCATATTGGGGTTGACCTGCAGTTTACTTGTGACTTTCTCTTTGATTCTTATAAGAAGAAAGCAAACCCAAGTTTGTGACCAGTGCAAAGAAACAGGAAATCCTACAACGTTCCTTCCTGCTGAACGAGACATATTACCAGAAACCCATTTGAACAATGAGGAAGTTGAAGGACCTGAAATAAACTATGCCGCATTGCACTTCTCACGGAAAACCAAATGGCCGAAGAAAAAGAGGAAGTTTAAGGAAGAATCCATATATTCAACTGTGAGACACTTCAGTGATGGAGAGGCTAATGAGGACTGA

>NITR23

ATGCTGATTTTATTTTATTTACTGTTGGTGTTTGGAACTGAGTGTTGTGCTAATGATTTATTCTATGAGACAAAGGCTGTGACAGTTGGAGATCATGTGAAGTTAAACTGTTCACGCGGGTCTGCAGGAGAGCTTGTATGGATGAGAATAGTTTCTGATAATCCTCCAGAAAATCTAACTCAAAATAAGACTCCTAATGTTAAAGTTAATCCAGAACCTGGATCGCTTGAACTGAAAATAACAGAAGCAAAGCTGAATGATACTGCACTTTACATTTGTATGAGGATAAAAGATGAACATCTTCTGTCTTTCAATGTGACATACTTGAGAGTTGAAGAACTTGCTGTGACGGAGGCCCCTCCATCTACTCCATCTATTCCTGTTTGCCCAAGAGACTCGCTGACTTTGCAATGTTCAGTTCTTCACAAATCCTTAAGGAATTCATGTCCATCCAATGAAAGCGCGTTCTGTTTCAGTGTTGATTTTAGTGAGCCTGACCCAAATAACACTAAAAATAACAGAGCAAATGAAGAGGAGAACACCTTTGAGGGAAATGTCATAACAAAATGTGCGTCCTTTTCAAACAACTTTATTTCTTCTGATGGTTGGACATATTTCTGTGCAGTTCCCAAATGTAAAGACAAAACACCAGTGGAAACATCTCAAATCAACACTGGAGCAAACATTTGGACTTCAAGGATGCAAGAAGTTGCCATAAAATGTTTAACTGCTGCTTTGTTCGTCAGTCACATAATTATAGTTTTCTTAATTTATTTAGTCAAGAAGCTGAAAAAATAA
