# Supplementary material for: The medaka novel immune-type receptor (NITR) gene clusters reveal an extraordinary degree of divergence in variable domains
Source: BMC Evol Biol. 2008 Jun 19;8:177. doi: 10.1186/1471-2148-8-177 (PMC2442602; doi:10.1186/1471-2148-8-177)
Supplement: Additional File 2 — Protein sequences encoded by predicted NITR transcripts based on the 1st draft of the medaka Hd-rR genome. Sequences are in FASTA format and include translated sequences from Additional file 1. Predicted protein domains are color-coded (leader = green; V domain = light blue; I domain = red; transmembrane domain = pink; cytoplasmic domain = yellow). [file 1471-2148-8-177-S2.doc]

>NITR1a

MVTPAQFVVMLTCLLSGNSAQSPPLGSSSSDHQESVFLSAHIGETVTLQCFYDGVFLQYILWYKHILGRKPKLIAYFTKYETPLKISHEYQNNPRFTLKSSQQSSNLTISDLKPSDSATYFCISDHQTYLFFKSAFTLDVKGSGLTIQTSVSQSSSENIHAGDSVTLNCTVHTGSCDEEHRVYWFKDSEDSHPGLIYTHGGRNDQCERKNNTQTHSCVYELHIKNLTESHAGIYYCAVVSCGHILFGNGTKLDLTAPLNSVILNTLVGLLTVMSVLVVFLLFLLWKIHKSNNCTSTEERSPAAPLRSSEAENKDTESLHYAAVNVKKSNRSRRQKNEAKTDYLYASVRQQN-

>NITR1b

MVTPAQFVVILTCLLSGNSAQSPPLGSSSSDHQESAFLSAHIGETVTLPCFYDGIYLRYILWYKHILGRNPTFIAYFTKYSPEKEISHEYQNNPRFTLKTSEQSSNLTISDLNLSYSATYFCISHHYTYLFSQAAFTLDVKGSGSTIQTSVSQSSSENIHAGDSVTLNCTVHTGSCDEEHRVYWFKDSEDSHPGLIYTHGGRNDQCERKNNTQTHNCVYKLSIKNLTESHNGIYFCAVVSCGHILFGNGTKLDLTDSAAPLNSVILNTLVGLLTVMSVLVAFLLFLLWKIHKSNNCTSTEERSPAAPLRSTEAENKDAESLHYAAVNVKKSNRSRRQNDNSNNDCLYASVRQQN-

>NITR1c

MVTPAQFVVLLTCLLSGNTAQSPLLGSSSSDHQESVFLSAHIGETVTLPCFYDRVYFRYILWYKHILGQKPKLVAYFTKYNPDIPIPHEYQNNPRFTLKTSDQSSNLTISDLNLSDSATYFCISDHLTHMNFSAVLTVNVKGSGLTIQTSVDQSSSENIHAGDSVTLNCTVHTGSCDEEHRVYWFKDSEDSHPGLIYTHGGRNDQCERKNNTQTHSCVYKLHMKNLTESHAGIYYCAVVSCGHILFGNGTKLDLTDSAAPLNSVILNTLVGLLTVMSVLVAFLLFLLSKINKSNNCTSTEERSPAAPLRSTEAENKDAESLHYAAVNVKKSNRSRRQNDNSNNDCLYASVRQQN-

>NITR1d

MGTPAQFVVILTCLLSGNTAQSPPLGSSSSDHQKSVFLSAHIGETVTLQCFYDGVYLQYILWYKHILGRKPKPISLFSKYSAELIYYNNYKNNPRFTLTTSDQSISLIISNLKQSDSAIYFCVAGYQTHMNFSAVLTVNVKGSGLTIQASVDQSSSENIHAGDSVTLNCTVHTGSCDEEHRVYWFKDSEDSHPGLIYTHGGRNDQCERKNNTQTHSCVFKLHIKNLTESHAGIYYCAVVSCGHILFGNGTKLDLTGSAVPVNSVILNTLVGLLTVMSVLVVFLLFLLWKINKSNNCTSTEERSPAAPLRSTEAETKDTESLHYAAVNVKKSNRSRRQKNDSNTVCVYASVRQQN-

>NITR2a

MTLVVFAGCVTCLLLGTVANSWAQESSASLHFESVFVGQEVTLKCFLEGTGANVFFWYKQPLGQKPQLMSEFFNHKEIGTFADDFKKDPRFELQTNEDKNNLKISNVKMSDSATYYCISSYTYTLTFLEAYSLHVRDTSSDIQTSVDQSSSENIHAGDSVTLNCTVHTGSCDEEHRVYWFKDSEDSHPGLIYTHGGRNDQCERKNNTQTHSCFYKLSIKNLTESHAGIYYCAVVSCGHILFGNGTKLDLTAQEGNLLPFVYFLSGALTASLIFLTSYAIHKTKSCKSKGVWGVKVPNTFLDRHQAVNFTSAKCEFDVRIALDVNPALWINSNNILCYVQRASSGLLEIRMLF-

>NITR2b

MTLLVFAGCVTCLLLGTVANSWAQKSSASLHFESVFVGQEVTLKCFHGGTVADFFFWYKQPLGQKPQRMSTFLDYNKNGTFLDDFKKDLRFELQTNKDTHHLKISNVKMSDSATYYCISSYSYAFTFLEAYSLHVKDHSYIQTSMDQSSSQNIHAGDSVTLNCTVHTGSCDEEHRVYWFKDSEDSHPGLIYTHGGRNDQCERKNNTQTHSCVYELPIKNLTESHAGIYYCAVVSCGHILFGNGTKLELTGQTNSLLVYFLTGTLTFMSILVVFLVYKIKKHICFQSKDEGSAAASTPNTEAENKDTESLHYAAVNVKKSNRSRRQKNDLNTVCVYASVTQQN-

>NITR2d

MTLLVFAGCVTCLLLGTVAYSWAQKSSASLHFESVLVGQEVTLKCFHRGTVADFYFWYKQPLGQKPQRMSEFFDYKKNGTFSDDFKNDLRLQLQTNEGKNHLKISNVKMSDSATYYCISSYTYTFTFLEAYSLHVRDTSSDIQTLVTQSSSENIHAGDSVTLNCTVHTGSCDEEHRVYWFKDSEDSHPGLIYTHGGRNDQCERKNNTQTHSCVYELHMNNLHEPGIYYCAVVSCGHILFGNGTKLDLTGQTNSLLVYFLTGSLTFMSILVVFLVYKIKRQICFQSKDEGSAAASTPQTEAENKDTESLHYAAVNVKKSNRSRRQKNGGNTDCVYASVRPQN-

>NITR3a

MVNLTLVLVLLCTLSLISLSTPEFHTVKVQPEGEVTLKCSNFSNFISYIVWFKLNDGRNATIISSMITSESNVSMKDGFKERFFMTSNITHVFLNIKNVNLSDSGLYFCGHRGSTSAVIFGATYLLVYEMSRSPDLQTVIVGSIIAFLLMVIIMLFMKIRSYPKASVERQLKESLDSDALNYAAVSFQWKAKTRINAAYTKVVYATTK-

>NITR3b

MVNLTLVLALLCTLSLISLSTPEFHTAKVQPEGEVTLKCSNFSNLISNILWFKLNDGRNATIISSMITSESNATMLDGFKERFFMTSNITHVFLNIKNVNFSDSGLYFCGHRGSTSAVIFGATHLLVYEMSRSPELLTIILGSIIAFLLMVIIVMLFMKIRSYPKAPVERHQKENLDSDALNYAAVSFRWKAKTRTKAAEDINVVYATTK-

>NITR3c

MVNLTLVLALLCTLSLISLSTPEFHTLKVQLEGEVTLKCSNFSDLISNILWFKLNDGPNATIISSMITSDSNASMKDGFKERFFMTSNITHVFLNIKNVNFSDSGLYFCGHKDGARAVIFGATYLLVNEMSRSAELQTIILGSIIAFLLMIIIVMLFMKIRSYPKAPVERQQKENLDSDALNYAAVSFRREAKNRTKAAEDINVVYATTK-

>NITR3d

MVNLTLVLVLLCTLSLISLSTPEFHTAKVQPEGEVTLKCSNFSNLISNILWFKLNDGRNATIISSMITSDSNASMKDGFKERFFMTSNITHVFLNIKNVNFSDSGLYFCGHRGSTSAVIFGATHLLVYEMSRSPNLQTIILGSIIAFLLMVIIVMLFMKIRSYPKAPVERQQKANLDSDALNYAAVSFRRKAKTRTKAAEDINVVSVRQLLQ-

>NITR4a

MISKRFVFFLTLLFVAANGQKTYPKASSSVQQKGKGFLSVSVGDTVTLECSYEGPNSVQIFWYKQSFGQKPKLMSTFYGHTTKETFSDEFKSNSRFKLETENQNPHLKISNLKFSDSATYHCISSDSYSLTFLESFRVLVKDPSSYVLTSVDQSSSENIHAGDSVTLNCTVHTGSCDEEHRVYWFKDSEDSHPGLIYTHGGRNDQCDRKNNTQTHSCVYELHIKNLTESHAGIYYCAVVSCGHILFGNGTKLDLTVPLNSVILNTLVGLLTVMSVLVAFLLFLLWKIHKSNNCTSTEERSPAAPLRSSEAENKDAESLHYAAVNVKKSSKTRRQKNNSNTVCVYASVRQQN-

>NITR4b

MISKLFVFFLTCVFIEANGQKTHLKLSSSEEQKQFFLSVDAGETVTLKCSYEGHTLRWIFWYKQSLGQKPKLLSSFYVHGTEVIFFDEFKNNSRFKLDTENKNFHLKISNLQFSDTATYHCIGSDSYSLTFVEGYSVHVKDRSFYLKFSSENIHAGDSVTLNCTVHTGSCDEEHRVYWFKDSEVSHPGLIYTHGGRNDQCERKKNSQTHSCVYELYMKNLTESHAGIYYCAVVSCGHILFGNGTKLDLTAPLNYVNLNTLVGLLTVMSVLVAFLFFLLWKIHKSNNCTSTEERSPAAPLRSSEAENKDTESLHYAAVNVKKSSRSRRQKNDLNTVCLYASVRQQN-

>NITR4c

MISKLFVFFLTCLFVGGNGLDMYPKILSSVQQKGDFLSAYVGETVTLECSYEVKTVKRIFWSKQRLRRKPELLSSFYVHSTEVTFFDEFKNKSRFRLDTENQNHHLKILNLQFSDSATYNCISSDSYSLTFLESFSVLVKDPSSYVLTSVSQSSSENIHAGDSVTLNCTVHTGSCDEEHRVYWFKDSEDSHPGLIYTHGGRNDQCERKNNTQTHSCVYELHMKNLTESHAGIYYCAVVSCGHILFGNGTKVDLTDSAAPLNSVILNTLVGLLTVMSVLVAFLLFLLWKIHKSNNCTSTEERSPAAPLRSSEAENRDAESLHYAAVNVKKSNRSRRQKNDINTVCLYASVRQQN-

>NITR5a

MTLLVFSGCLTCLLLGTVALSWAQKPSASLQFQSVHVGDEVTLKCIRQGTGIENIYWYKQPLGMKPQLMSEYLDIKKNGYFIDAFKNDPRLKLETDKNKHHLKISNLKMSDSATYYCISSDFYGVKHLEGYTVHVKDSTSDIHASVDQSSSENFHAGHSVTLNCTVHTGSCDGERQVYWFKDSGDSHPGLIYTHGGRNDPCGRKNNTQTHSCVYELHMERLTESHAGIYYCAVVSCGHILFGNGTKLDLTGSFPPFVYFLSGALAASLIFLTSYAIHKIRNHKCRARNPQDRSAVDSGPDAEGTEDLHYAALRHSKLNKS-

>NITR5b

MTLLVFSGCVTCLLLGTVALSWAQKPSASLQFQSVLVGEEVTLKCNRQGTGTDIIYWYKQPIGLKPQLMSEYLDFRKNGSFVDAFKNDPRLKLETDKDQLHLKISNLKMSDSATYYCISSDYYGVKHLEGYTVHVKDSTSDIHASVDQSSSENFHAGHSVTLNCTVHTGSCDGEHRVYWFKDSGDSHPGLISTHGGRNDQCGRKNNTQTHSCVYELHMERLTESHAGIYYCAVVSCGHILFGNGTKLDLTGSFRPFVYFLSGALAASLIFLTSYAIHKIRTHKCRARNPQDRSAVDSGPDAEGTEDLHYAALRHSKVYKSRREGRCRQ-

>NITR5c

MTLLVFSGCVTCLLLGTVALSWAQKPSASLQFQSVLVGEEVTLKCNHQGTGADIIYWYKQPLGLKPQLMSEYLDFRKNGYFIDAFKNDPRLKLETDKDKHHIKISNLKMSDSATYYCISSDFYGVKHLGGYTVHVKDSTSDIHASVDQSSSENIHAGDSVTLNCTVHTGSCDGEQRVYWFKDSADSHPGLISTHGGRNDQCGRKNNTQTHSCVYELHMERLTESHAGIYYCAVVSCGHILFGNGTKLDLTDSAAPLNSVILNTFVGLLTIMSVLVAFLLWKIKKSNACTSTEERSPAAPLRSTEAESEDAENLHYAALNVKKSKKTRRQENDLNTVCVYVIVRQQN-

>NITR7a

MISNHFVVFLTCLSFGAKAQKNVLESSSVIQKGGFLSVSTGEAVTLECSYKGLDLTWIFWYKQTLGQRPEPISSFYTYASVLTFFDPFNNNPRFTLDTKNQNHHLRISDLQLSDSATYYCAARHATIVTFTEGVTVRVKGPGVTTKTSVDQSSSENINAGDSVTLNCTVHTGSCDGEHRVYWFKDSADSYPGLIYTHGGRNDQCGRKNNTQTHSCVYELHMERLTESHAGIYYCAVVSCGHILFGNGTKLDLTDSAAPLNSVILNTFVGLLTIMSVLVAFLLWKIKKSNACTSTEERSPAAPLRIIEAESKDAENLHYAALNVKKSQKSRRQKNDLNTVCVYASVKQQN-

>NITR8a

MILFYVVFIITNGYCANEYHFTTKTVPVGEDVKLTCARQTNVLYRENLFWIRIVSGKKPELLGGTMNFDFDDEIRKSHITAKQEPGSFVLEINGAMESDDGVYYCIKVQNLDLTFLTGTFLSVKGREPHIVAVTERFLSDQVYPEDPITLECSVLSSSDHETCAAEQRVFWFKTQSNKSHPHVIYAHGNSSDECLRTPEAPSVQKCVYSFNKNFISSDAGTYYCAVAACGEIFYGNGTTLTEPQMWDLQTANTVLLVLFATFSASIFVIIFLLYKIKKKSGSSCNDILHSLDDQNHQKSKNDSLTYSAATFTKREAGRFATSQEAL-

>NITR8b

MILFYVLFIITNGYCANEYHFTTKTVPVGEDVKLTCARQTDVLYRENLFWIRIVSGKKPELLGGTMNFDFDDEIRKSHITAKQEPGSFVLEINGAMESDDGVYYCIKVQNLDLTFLTGTFLSVKGREPHIVAVTERFLSDQVYPEDPITLECSVLSSSDHETCAAEQRVFWFKTQSNKSHPHVIYAHGNSSDECLRTPEAPSVQKCVYSFNKNFISSDAGTYYCAVAACGEIFYGNGTTLTELQMWDLQKANTVLLVLFATLSASIFVIIFLIYKMKRKAGTTSNDGLHSSDDRKHEKNKEDSLTYSTPTFTQRKAGKAKRKQKTPQETFYSDIKNLG-

>NITR9a

MQLRVILCGLFHLSAVIWAGAVKQDTGVRSVSVGENVTLQCFYENVMAMHFSWYQQPLGGRPELLSFFYKYDNPSKVDHWLQKKPRFSLQREEGINHLHISDVQLSDSATYFCGSSHSNMVEFGDGLFLSVEEKSPTEIIIQEPTSETIQPGGSITFSCTVHSGNCGEAQTVCWFRRGSQPGVLHTQRKDCRPVAAPGPPSQSCTYSLQKKDLNSSDAGTYFCAVASCGKMLFGSGTKLIMTNQTEGQAAQIKVLVQLSVIRTGVLLLFLLSCVLFVRKSDPSP-

>NITR9b

MQLRVILCGLFHLSAVIWAGAVKQDTGVRSVSVGENVTLQCFYENVMAMHFSWYQQPLGGRPELLSFFYKYDDPSKVDHWLQKKPRFSLQREEGINHLHISDVQLSDSATYFCGSSHSNMVEFGDGLFLSVEEKSPTEIIIQEPTSETIQPGGSITFSCTVHSGNCGEAQTVCWFRRGSQPGVLHTQRKDCRPVAAPGPPSQSCTYSLQKKDLNSSDAGTYFCAVASCGKMLLGSGTKLIMTNQTEGQAAQIKVLVQLSVIRTGVLLLFILSCVLFVRKSDASP-

>NITR9c

MQLRVILCGLFHLSAVIWAGAVKQDTGVRSVSVGENVTLQCFYENVMAMHFSWYQQPLGGRPELLSFFYKYDDPSKVDHWLQKKPRFSLQREEGINHLHISDVQLSDSATYFCGSSHSNMVEFGDGLFLSVEEKSPTEIIIQEPTSETIQPGGSITFSCTVHSGNCGEAQTVCWFRRGSQPGVLHTQRKDCRPVAAPGPPSQSCTYSLQKKDLNSSDAGTYFCAVASCGKMLLGSGTKLIMTNQTEGQAAQIKVLVQLSVIRTGVLLLFILSCVLFVRKSDASP-

>NITR10b

MHVVLCSLLILQLGCCADEGFETKTVDSGEDVTLLCNNSRLNFGFFFWMKSVPGQMPEIVGKRFGKSDEFNKIHHFTTKEEDGRFFLQISEAKPSDSAFYYCFTFKNYKITFMKAVLLRIKGPRSDFPAVVQSSVDLDSAGDVVALQCSVLSEFKNDACPEEQRVFWFRKTEGESHPTYIYARRSSDGDCDGGTETQPLQSCVYSFLKNVSSSDGGLYYCAVAACGKVVFGNGTKLDVQVDGTYDSRNNNRYFSLFVGTLVLSLTMVTFLIWVVSKKSCDVCKVYLACKATSDTVNDEQQSQKINQNNVVYAATVFAKKKADKTKGKKRSNKGRDHLQ-

>NITR11a

MRSLIVVTTFLLCSRSCFCESKTVEVQSGENVSLLCSDFTKNRQQTDWFRVVKSSKVSCISSMFGVDGDPSFCDGFDGGKFEMNSNSSSVSLKISGVDESDSGLYFCGFYRNRHTVIGDVTQLIIKDSTVFMKHPYTSLLTVILAVLMSFFIFGALGLVVIFEHHCAAKENLHTDSLKTQDSSDPNSAALKFSKSRRSGRPAESRQLETCVMYSVRRQTYSSV-

>NITR11b

MWSLAIITVFLLCSRSCFCESKTVEVQSGENVTLLCSDFTKNRQQTDWFRMVNSSKVSCISSMFGVDGDPSFCDGFDGGKFNMSSNSSSVSLKISGVDESDSGLYFCGFYRNRHTVIGDVTQLIIKERHVRMDLLSVLLAALTVLLSAVVVVLAVKIRKLLTAARKEQENTKNLDSNDLNYAALSFNQKPKKGGRSPSDRELQPHVLYAPTR-

>NITR12a

MGLTSILSFLCSFSLICVSLSEFVVVEVQLGGEVSLLCSNLSNIMSNIFWFKSAKRSNTTRIASMPTAESNATVLEDFKNGRFHMSSNTTHVFLNIKEFNISDSGLYFCGLNTINYNFDATFLQVEETPAFNLVVGILGSVIFVLVMVIIFLPVKIKSFQKAPSERQHRESLESNVRYYAALTFQPKPETQSISVYKTDSDLCFWS-

>NITR12b

MGLTSILSFLCSFSLICVSLSEFVVVEVQLGGEVSLLCSNLSNIISNIFWFKSAKRSNTTRIASMPTAESNATVLEDLKNGRFHMSSNTTHVFLNIKEFNISDSGLYFCGLNTINYNFDATFLQVEETPAFNLVVGILGSVIFVLVMVIIFLLVKIKSFQKALPERQHRESLESNVRYYAALTFQPKPEIQSISVYKTDSDLCFWS-

>NITR13

MISKLFVFFLTCVFVGADGQKTYLKSSSFVWQKSGFLSVNVGDTVTLECSYEGYELTWISWYKQSLGGKPELMSSFYAYSTEVTFFDEFKDNSRFKLDIENKNHHLKILNLQFSDSATYYCAVSYAMILKFTEGVTVNVKGSGLTVMSSVDQSSSENIHAGDSVTLNCTVHTGSCDEEHRVYWFKDSEDSHPGLIYTHGGRNDQCDRKNNTQTHSCVYELPIKKATESHAGIYYCAVVSCGHILFGNGTKLDLTGDDDSDSFHTPFLVYLFTSLIVIICLLVLVIFLLCKMRQSNKYPSTEERSATLSLTSTKVKVQEAESLQYATVDVKRMNRSKRQKNDTSSQCIYTSVREKS-

>NITR14

METSAHIVFFLTLLLSVQMTLLASSSSVHADSEFVSGLIGQTVTLPCDYNRSDFIWVAWYKQMVGKKLDLISQMFRYQKTVMKNPRYSVKSQNQKYHLNILNLQDSDSATYHCTICFAASIDFLNVVTVNVKGSGSTIQTSVHQSSSENIHAGDSVTLNCTVHTGSCDEEHRVYWFKDSEDSHPGLIYTHGGRNDQCERKNNTQTHSCVYELYMKNLTESHARIYYCAVVSCGHILFGNGTKLELTDSSAPLNSVILNSLVGLLTVMSVLVAFLFFLLWKNHKSNNCTSTEERSPAAPLRSSEAENKDAESLHYAAVNVKKSSRTKSQKNDSNTDCLYASVRQQN-

>NITR15

MKTSAQFVLFLTVLLSVGKILLTSSSLLHQQTDFKSAHKGETVFLPCIYEVDDLIWVSWYKQALGQKPKLVSRFFRYRKQPNFFHDFNDNPRFLLHTENQNNHLTILDLQYSDSATYLCVANFGKDLNFSSAFTIDVKGSGLTIQTSVSPSSSENIHAGDSVILNCTVHTGSCDEEHRVYWFKDSEDSHPGLIYTHGGRNDQCERKNTQTHSCVYELHMKNLTESHAGIYYCAVVSCGHILFGNGTKLDLIDSAAPLNSVILNTLVGLLTVMSVLVVFLLFLLWKINKSNNCTSTEERSPAAPLRSTEAENKDTESLHYAAVNVKKSNRSRSQKNDINTDCVYASVRQQN-

>NITR16

MVTPAQFVVILTCLLSGNTAQSISLGSSSSVLQESVFKSAHIGETVTLPCFYKEFYLKYVSWYIYILGKKPKQLSYLRKYDKNVTLSDEFKNRFTLKPGNQSSHLIISDLKQLDSATYFCIAGYQTHLTFTAAFTVDVKGSGLTIQTSVDQSSSENIHAGDSVTLNCTVHTGSCDEEHRVYWFKDSEDSHPGLIYTHGGRKDQCERKNTKTHSCFYKLYTKNLNRTHAGIYYCAVVSCQPNSLLVYFLTGSLTFMSIVVIFLVYKITKDICCQSKDQISAAGSTPQTEIESEDTESLHYAAINVKRSNKSRRQNGSITDCIYASVTQQN-

>NITR17

MASIPCAIFLTVLFSGEMAQTKTSSLSSSLRHDTDFLTVKPGDNLSLKCFYEDVVDARFYWYKQSLGQKPKLISISYKYENKGVFHGEFRNSSRFTLDNRKGNNHLMIANLHYSDSATYYCASSYLYNFEFSDGITVNVKGSDLTIQTSVDQSSSENIHAGDSVTLNCTVHTGSCDEEHRVYWFKDSEDSHPGLIYTHGGRNDQCERKNNTQTHSCVYELPDLTESHAGIYYCAVVSCGHILFGNGTKVDLTAEVPYQNYLWSGALFFTTLLCVFLGISLCLMTRSHNRKMSGSSQPPKGAMGFKSTEKVYHAAVCTNLANRSRRLRDPTWSECVYNSMKQ-

>NITR18

MIRGLAALILLNLLTVVQNLELLQQISAELGDNVTLTCLTSGVDHGLFFWYKFQFGYRFQMVSSGNFGQLKLEQQFDTPRFNMVNVGNIYSLNIRNISKEDEGMYICQAGAAYKLRFISGNRLMVKDPKKRPKTIFVNQSSIMEAVLSGQSVNLFCSVLLNTRENSDLCSGKHRVYWYRAGSESHPHLIYTTRSSCDVQKGMRCIYNLTQTIRGLSDSGVYYCAVVLCGEILFGEGTKVQIKQLSPDAIVLGSLLACCVLMNITLIVMRTKQKCEQCKDSYYILVMLFYFWVTCTNDGEEDGQSGQRAIASQHNCLDLNKRNNLKSKLPGTLTTHPFINQMKK-

>NITR19

MSVRLAACLFLCVVAVPQTLQLSQKISITEATFGENVIMKCTTVGLEQRMVYWHKLQFGFMIQTIAFGSSPDLPLKEGFNNSRYSAKKEGNEYSLEIRNVSKEDQGTYFCQAGTSYTMTFIYGSHLVLKGSNLESVSPGSKVNRQCSLLWQPEKNPDRCLGEQRAYLYRAGFESEPDIIYTTSSRCDDQEDKSCVYHVSKPMKNSLDPGLYSCAVSSCREILFGEDTEEQIRQGVCPYVLILGTLLACCVLANIILIVTWKKQNPVCDKCKDGKAHTNAENDGPAGDQQADLGEEDDGLHYVALEFSSRKSKRQRSMRRESKETCIYANKRDY-

>NITR20

MIRGLATFILLGGVFVIETLELPEEIPLIEAELGDNVTFTCSSQDFDQALMYWYKFQYGYVIHTILKSSFGKFNEHFDDSKIYTVKKNDDKLILTIKNVSKEDEAAYFCQAGSSYNMEFINGIHLFVKGPKNELKSGSVKPSPKLELVLLGNTVNLQCSVLSEKNLCARERRVYWYKAGSKSPADIFHATSPSCDDREGRCVYNLSKTIQTFSDCGVYTCAVVSCGEIMFGEGTKVQMAQDMLCTNAKQHISIFGTLLACSILANIALFLARKRQKGVFGLCKGDAARSSQAEQLRSAEDQPNHGGFGETEMCYAALDFTPGQLRRPKITENPAEDYIYSQTMFKRRVREE-

>NITR21

MIGELSAFILLTAIFVTQPMETPSKISLTKAELGDDVTLICSTTGIDNNLLSWFKYELGYVIKTVGKINYGIVQMYGQFNSSRFGIIPEAKGGSLSISNVSQEDEGTYLCQAGSYLTMRFFNGSHLVVKDPTKKQNAVHVQQSSDAESVFQGNTFNLQCSVQSKIKKNPDPCLGEHRVYWYQAGSETHADIIHATSPSCDDHGRRCVYNLSKTLQNFSDSGISSCAVLSCGQILFGEGNKVRMKQPEFLYLLILGTLLVCSVLVNTALIVIVGKGCCEHSKDAAAPRHRKREKSAEDDTNLDDEEAELNYAALRFPPKTKNLRRTNEFTEDNF-

>NITR22

MQGGQITFILLCALSVTQSLAPELVHMIEAETGSNVTLKCSASGTDQQLFFWHKLQFGYMIQSVATGNMANIALKKNVERSRFNVTKVGDVYSLFIRNVSKEDEAMYLCQAGASYAAKFTNSSQLVVKGPKKKQKFVKQSPDVELVLLGNTVNLQCSVLLNTRESSDECSDDHRVFWYKAGSEKHADNIYITNSGCDVQKERRCVYNLPKVVKNSSDSGVYYCAVLSRGEILFGNGTEVLIKKELCPYVIILGLTCSLLVTFSLILIRRKQTQVCDQCKETGNPTTFLPAERDILPETHLNNEEVEGPEINYAALHFSRKTKWPKKKRKFKEESIYSTVRHFSDGEANED-

>NITR23

MLILFYLLLVFGTECCANDLFYETKAVTVGDHVKLNCSRGSAGELVWMRIVSDNPPENLTQNKTPNVKVNPEPGSLELKITEAKLNDTALYICMRIKDEHLLSFNVTYLRVEELAVTEAPPSTPSIPVCPRDSLTLQCSVLHKSLRNSCPSNESAFCFSVDFSEPDPNNTKNNRANEEENTFEGNVITKCASFSNNFISSDGWTYFCAVPKCKDKTPVETSQINTGANIWTSRMQEVAIKCLTAALFVSHIIIVFLIYLVKKLKK-
