# Supplementary material for: The medaka novel immune-type receptor (NITR) gene clusters reveal an extraordinary degree of divergence in variable domains
Source: BMC Evol Biol. 2008 Jun 19;8:177. doi: 10.1186/1471-2148-8-177 (PMC2442602; doi:10.1186/1471-2148-8-177)
Supplement: Additional file 3 — Tetraodon nigroviridis encode multiple NITRs. Putative NITR genes and gene transcripts were identified from Tetraodon nigroviridis by a tBLASTn search using a Spheroides nephelus NITR1 protein sequence [GenBank:AAF28781] as a query. This strategy identified six candidate Tetraodon cDNAs [GenBank:CR683680, GenBank:CR675622, GenBank:CR664265, GenBank:CR725053, GenBank:CR724466, and GenBank:CR707240] and a single genomic BAC clone B22K12 [GenBank:BX629355]. Only one of these cDNAs [GenBank:CR683680] appears to include an entire ORF. The partial protein sequences encoded by [GenBank:CR675622] and [GenBank:CR664265] are identical. The partial protein sequences encoded by [GenBank:CR725053] and [GenBank:CR724466] differ at three predicted amino acids residues; [GenBank:CR725053] is 10 amino acids longer at the amino-terminus. BAC B22K12 encodes 12 different NITR genes (designated by their nucleotide location in the reverse complement position within BAC B22K12). The predicted protein sequences of four representative NITR cDNAs are aligned relative to the predicted protein sequences of the NITRs encoded by BAC B22K12. Note that NITR-116851 (BAC B22K12) is predicted to encode a V domain and lacks an I domain. [file 1471-2148-8-177-S3.pdf]

```
cDNA_CR683680    1 -------MALLCILLALFSEFAPLAAKSSGVNQDPRVLMAQVGQTVTLKCFCGEDSVTFFFWYQQRLGGKPVIVSSRMRHNVEVSVSPSFKG--RFEVES
cDNA_CR675622    1 QDAKHVHTNYCYSISCFLLHSVLTAGASSFVRPADGLVFVVAGGYIELQCSCQSESMTAFHWFKQSLGEKPSLIATVYKHNPTLQMSKDFENNHRFSVNL
cDNA_CR664265    1 QDAKHVHTNYCYSISCFLLHSVLTAGASSFVRPADGLVFVVAGGYIELQCSCQSESMTAFHWFKQSLGEKPSLIATVYKHNPTLQMSKDFENNHRFSVNL
cDNA_CR725053    1 -----------------------------------ITDKAGQNLTLTCFYNDDVSTDIYWFKQSLGEKLVVLRSYFISTQSSSFFNNFKNNPRFQVHL
72801            1 -------MALLCILLALFSEFAPLAAKSSGVNQDPRVLMAQVGQTVTLKCFCGEDSVTFFFWYQQRLGGKPVIVSSRMRHNVEVSVSPSFKG--RFEVES
77051            1 ---MESVTLASYLLCFCLWSLVLTAGASSFVRPADGLVFAVAGGNIELQCSCQSESETAFHWFKHSLGEKPSLIATVYKHNPNVQVSKDFENNHRFSVNL
81391            1 ---MVSLNEDSYLSCFCLWSLVLTAEASLSPQPVSGLIFANVGDKMTLPCSREDNSKNQFHWYKQTLGGKPRRMSSIYKHARNIEFKEEFQSDIRLSLNI
85651            1 ---MVSLNEASYLSCFCLWSLVLTAEASLSPQPVSGLIFANVEDKMTLPCSREDNSKNQFHWYKQTLGGKPRRMSSIYKHARNIEFKEEFQSDIRLSLNI
89901            1 ---MVSLNEASYLSCFCLWSLVLTAEASLSPQPVSDLIFANVGDNMTLPCSREDNSKNKFDWYKQTLGGKPRLMSSFYEHDTNVKFKEEFQSDIRLSVNI
96001            1 ---MVSLNEASYLSCFCLWSLVLTAEASLSPQPVSGLIFANVGDKMTLPCSREDDSKNQFHWYKQTLGGKPRRMSSFYKYDTNVEVKKEFRSDIRLSLNI
99501            1 ---MVSLNEASYLSCFCLWSLVLTAEASLSPQPVSGLIFANVGDNMTLPCSREDDSKNQFHWYKQTLGGKPRRMSSFYKHDTNVEFKEEFQSDIRLSLNI
104101           1 ---MTFAKFVFFLLCVEKLAQATTTDFS---QLGNRRISVKVGQNLTLTCFDNDAASTRIFWFKQPQGEKPSLLCLYYPSSQSTKCSKNFENNPRFQLHS
109901           1 ---MASVKLIFFLMCLEKIAKTATMEFSSSINWKNTFIHVKPGQNLTLPCLYTGTVYARISWFKQTVGEMPKLICVYRTSSQTWTFYHDFKSNTRFNLHP
113101           1 ----MTPAFIFLLMCLEKIGQAATMEFLPSMNWKNNFIVVKAGQNLTLPCLYREKFNTRISWFQQTLEKKPTIICVYRISSGNLAFYHHFDNNPRFKLHP
116851           1 ------------MTNIALITALLSTFISFSGCVFHTLSVRRGDNVTLQCANFTSMMFHVVWFKMNDRHNASSIASMRSSDSNVTLYEGFESGK-FNMSS
124301           1 ---MTSVTFFFFLMCLEKIAQAATTEFSPSMDWKSSFILVKPRQNLTLSCLYRDIIYTQISWFKQSLGENPRLICVYSISSQSQSFYNDLKNNQRFNLYP

cDNA_CR683680   92 REGMNHLTISDLLPSDSAVYYCGILEFNLLQFGEGTFLHVRTPLLNVRAVAYQPGLSSVPPGDSLELSCEVRGEKCKGEQSFYWFKQTTAQPVILHPIEG
cDNA_CR675622  101 TQDSCPLTIANLSISDSATYYCYSSYMYEFQLDKVYIVHVTSLHLNTPVEIQQSPIESLQAGESGILNCTVHTGTCDEELSVYWFRTSGASEPELIYAHR
cDNA_CR664265  101 TQDSCPLTIANLSISDSATYYCYSSYMYEFQLDKVYIVHVTSLHLNTPVEIQQSPIESLQAGESGILNCTVHTGTCDEELSVYWFRTSGASEPELIYAHR
cDNA_CR725053   64 DDKLANLTIADVEFSDSAVYYCAKQYSYIFEFIQGYDVIVE----ASELTVNQLASESIKSGGSMTLNCAVHTGTCDEDHTVYWFRNSGRSQMELVYSQK
72801           92 REGMNHLTISDLLPSDSAVYYCGILEFNLLQFGEGTFLHVRTPLLNVRAVAYQPGLSSVPPGHSLELSCEVRGEKCKGEQSFYWFKQTTAQPVILHPIEG
77051           98 TQGGCPLTIANLSISDSATYYCYSSYMYEFQLDKIYIVHVTSLHLNTPVEVHRLPSESLQAGESGILNCTVHTGTCDEELSVYWFRTSGASEPELIYAHR
81391           98 TGRNYHLTIGNLSFSDSATYFCASSFVYTFQLDKIYIVHVTSLHLNTPVEVHRLPSESRQSGESGILNCTVHTGTCDEEVSVYWFRSSGGSEPELIYAHR
85651           98 TGRNYHLTIGNLSFSDSATYFCASSFVYTFQLDKIYIVHVTSLHLNTPVEVHRLPSESRQSGESGILNCTVHTGTCDEELSVYWFRSSGASEPELIYAHR
89901           98 TGRNYHLTIGNLSFSDSATYYCASSYAYEFQLDKIYIVHVTSLHLNRPVEVHRLPSESRQSGESGILNCTVHTGTCDEELSVYWFRTSGASEPELIYAHR
96001           98 TGRNYHLMIGNLSFTDSATYYCTSSYAYTFQLDKIYIVHVTSLHLNTPVEVHRLPSESRQSGESGILNCTVHTGTCDEEVSVYWFRTSGGSEPELIYAHR
99501           98 TGRNYHLTIGNLSFSDSATYFCASSYVYTFQLDKIYIVHVTSLHLNTPVEVHRLPSESLQSGESGILNCTVHTGTCDEEVSVYWFRTSGGSEPELIYAHK
104101          95 DNKGVNLTISHLEFSDSADYYCANQRLNSFEFIYGYNVLVE----DSELPINQSPSWPNLSGDSGTLNCRVHTGTCDEDHTVYWFRNSGPSQLELVYSHS
109901          98 HSKGINLTFANLNFSDSATYYCANQDQYFLEFIHGYNVIVE----DSELTITQSPSESIQPGGSGTLNCLVHTGSCDMNHTVHWFKHSGPSQLELVYSHN
113101          97 HSKGANLTITNLRFSDSATYYCANQDFYVCEFTVGYTVIVE----GSELSVNQSPSDSFQSGGSGTLNCTVHTGTCDRNHTIYWFRNSKKSELKLAYSHQ
116851          87 DSVTLTLTIKQVDSSDSGLYFCG---------------------------FKLQDYAVIQGGTSLIVEEEATTGLTVMHLIVGIQTTVLIGVIAALAVK
124301          98 HSKGVNLKITDLKLSDSATYYCAKRYFYKFDFTEAYNVNVE----GSEMTINQSASESIPSGGSVTLNCSVQTGSCDGDHTVYWFRDSGPSRLGLINNHK

cDNA_CR683680  192 KCVNEARGKK-------CTLTYSIHSANSSDEGVYYCALASCGKIKFGNGTQVQLTTKGSNAQALLVYGFSAMLAVSIAVLFALAFTAGTMKNQICSVCK
cDNA_CR675622  201 ARNDHCKGKK-----NTCFYNLSTKNLTP-KAGTYYCAVAACGRILFGGGNKLDNEHKVTG-ADYLLYFLTGALVFSTILCVLLASFLFKLHKRSSSQRP
cDNA_CR664265  201 ARNDHCKGKK-----NTCFYNLSTKNLTP-KAGTYYCAVAACGRILFGGGNKLDNEHKVTG-ADYLLYFLTGALVFSTILCVLLASFLFKLHKRSSSQRP
cDNA_CR725053  160 GRNNQCEKKT-----NTCVYSLFVKNVNISQTAIYYCAVTACGRIAFGGGN-------------------------------------------------
72801          192 KCVNEARGKK-------CTLTYSIHSANSSDEGVYYCALASCGKIKFGNGIQVQLTTKGSNAQALLVYGFSAMLAVSIVVLFALAFTTGTMKNQICSVCK
77051          198 ARNDHCKGKNNTCFFQICLFNLSIKNLTS-KAGTYYCAVAACGRILFGGGNKLDNEHKVRQSADYLVYFLTGALVFSTILCVLLASFLFKLHKRSSSQRP
81391          198 ARNDHCKGKN-----NTCFYNLSTKNLTS-KAGTYYCAVRLCGRILFGGGTKLENENKVRQSADYLVYFLTGALVFSTILCVLLASFLFKLHKRSSSQRP
85651          198 ARNDHCKGKN-----NTCFYNLSTKNLTS-KAGTYYCAVRLCGRILFGGGTKLENDNKVRQSADYLVYFLTGALVFSTILCVLLASFLFKLHKRSSSQPP
89901          198 ARNDHCKGKN-----NICFYNLSIKNLTS-KAGTYYCAVRLCGRILFGGGNKLENENKVTG-ADYLVYFLTGALVFSTILCVLLASFLFKLHKRSSSQRP
96001          198 ARNDHCKGKN-----NTCFYNLSTKNLTS-KAGTYYCTVRLCGRILFGGGTKLENENKVTG-ADHLVNFLTGALVFSTILCVLLPLFLFKPHERSSSQRP
99501          198 SRNDHCKGKN-----NTCFYNLSTKNLTS-KAGTYYCAVRLCGRILFGGGTKLENENKVTG-ADYLLYFLTGALVFSTILSVLLASFLFKLHKRSSSRLT
104101         191 SRNNQCEKRT-----KTCFYS--LKNVNISQTGTYYCAVAACGRILFGNDTRLNCEG--N---LVWVYFLSAAWILATIVIVSLS-ISIFMAKKRNSHPS
109901         194 KSRNECGEKT-----NTCFYSLNIKSWNSSHRGIYYCDVEACGRFLFGNGTKLDFED--KENLLVWVYFLSTASLFMMIMFILLS-IYTFKMKKRNSH--
113101         193 GRNNQCEKKT-----NTCFYSLSIKNLDISQTGTYYCAVEACGRILFGNGTKLEFED--KGNLFVWVFYLSAAWIFTIIVVVVLLSIYIFLTKKRNSP--
116851         159 IRRLQKDKAT--------RTNEDLDSDIQNYASLDFQEAKRQRRISPENPS-------------------------------------------------
124301         194 GSKNQCEKTT-----NTCFYSLSLKNLNISQTGTYYCAVAACGNILFGNGTKLDFEE--KGNLLVWVYFLSAAWIFTIIMAVLLF-ISILMMKKKNSH--

cDNA_CR683680  285 GTVAHQS-------PSACDPTDAGDVRYAAVELKKSSKHDSAEDENQCQESLCVYLKIN------
cDNA_CR675622  294 DIQTDPS------LEMSEMQIQEDNVHYAALKNHKVHKSRRKRESSGNNCVYSSVVTLQAS----
cDNA_CR664265  294 DIQTDPS------LEMSEMQIQEDNVHYAALKNHKVHKSRRKRESSGNNCVYSSVVTLQAS----
cDNA_CR725053      -----------------------------------------------------------------
72801          285 GTVAHQS-------PSACDPTDAGDVRYAAVELKKSSKHDSAEDKNQCQESFCVYLKIN------
77051          297 DIQTDPS------LEMSEMQIQEDNVHYAALKNHKVHKSRRERESSGNNCVYSSVVTLQAS----
81391          292 DIQTDPS------LEMSEMQIQEDNVHYAALKNHKVHKSRRERESSGNNCVYSSVVTLQAS----
85651          292 DIQTDPS------LEMSEMQIQEDSVHYAALKNHKVHKSRRKRESSGNNCVYSSVVTLQAS----
89901          291 DIQTDPS------LEMSEMQIQEDSVHYAALKNHKVHKSRRKRESSGNNCVYSSVVTLQAS----
96001          291 DIQTDAS------FEMSEMQVQKDNVHYAALRKRKVNKLGWKRESTESECVYSLFSDGSNELQFF
99501          291 ETQSGPS------FNMTEMQVQEDNVHYAALRDHKINRPRKKRPS--NDCVYASVHQ--------
104101         278 LDKESQSRFLAESTASTEGDQGEVNLHYAALRINRPNKPRTQR-NSQNKCVYSGVRQ--------
109901         284 HLLDSQLRFPVGSTANTEGDQEEANLHYASVTGSQKTRSRMQR-NIQNECVYSGVMG--------
113101         284 YLSDSQSSFPVAFTATTERNQEEDNLHYAAVRANQLSRSRIQR-NGHNECVYSGVRQ--------
116851         202 -----------------------ETNVVYATTR--------------------------------
124301         284 HLLDTHSRLPVAPTVNTEGDQEEDRLHYAAVRGNDYNRTRRQR-NRQNECVYSECVYSGVKQ---
```
