# Supplementary material for: The medaka novel immune-type receptor (NITR) gene clusters reveal an extraordinary degree of divergence in variable domains
Source: BMC Evol Biol. 2008 Jun 19;8:177. doi: 10.1186/1471-2148-8-177 (PMC2442602; doi:10.1186/1471-2148-8-177)
Supplement: Additional File 4 — Oligonucleotide primer sequences used for nested 3' RACE. Predicted translational start sites are underlined. Primers designed to amplify NITR10 amplified NITR24 transcripts. Primers designed to amplify NITR13 amplified NITR4 transcripts. Primers designed to amplify NITR16 amplified NITR1 transcripts. [file 1471-2148-8-177-S4.pdf]

Gene	Primary Primer Sequence	Nested Primer Sequence
NITR1	CGCCAGAAATCCAAACAGGKGACACC	ATGGKGACACCTGCTCAGTTTGTSGT
NITR2	AGCAGGTTCAGAAAAATGACTCTTST	ATGACTCTTSTSGTGTTTKCTGGCTG
NITR3	TYTGCTGTGCANACAATGGTGAACCT	ATGGTGAACCTTACCTTGGTTCTGGYT
NITR5	CAGCCGTTTTAGAACATGACTCTTCTG	ATGACTCTTSTSGTGTTTKCTGGCTG
NITR6	AGAGGCTKTGCCACAATGAGAGGACT	ATGAGAGGACTGGCTCTYTTWATCCT
NITR8	GACCAATACAAAATGATGATTCTGTT	ATGATTCTGTTTTATGTASTTTTYAT
NITR9	GAGAGCTCTGAGAAGATGCAGCTGCG	ATGCAGCTGCGTGTGATCCTCTGTGG
NITR10	TAAGAAGTTCAGAAAATGCATGTTGT	ATGCATGTTGTACTTTGTTCACTKCT
NITR11	CWGTTYARSACAGAATGWGGAGCCTC	ATGWGGAGCCTCRYCRTAACARYTT
NITR12	ATTGAGTACAGAATGATGGGCTTGAC	ATGGGCTTGACCTCAATACTGTCTTT
NITR13	AGAATCTGGACATGATGATTTCTAAGC	ATGATTTCTAAGCTCTTTGTTTTCTT
NITR14	ACAAAAATCCAAACATGGAGACATCTG	ATGGAGACATCTGCTCACATTGTTTT
NITR16	GCCAGAATCCAAACATGGTGACACCT	ATGGTGACACCTGCTCAGTTTGTCGT
NITR17	AAAATCTGAAGACTATGGCATCCATA	ATGGCATCCATACCATGTGCGATCTT
NITR18	GAAGGAAATGCAGCAATGATCAGAGGA	ATGATCAGAGGACTGGCTGCTTTGAT
NITR20	TTTGATGTGCCACAATGATCAGAGGG	ATGATCRGAGRGCTGGCTRCTTTCA
NITR22	GTTTTTGTGTCACAATGCAAGGAGGA	ATGCAAGGAGGACAGATCACTTTCAT
NITR24	AGAAGTTTAGAAAAATGCATGTCGTA	ATGCATGTCGTACTTTCCACACTACT
