# Supplementary figures and images for: The medaka novel immune-type receptor (NITR) gene clusters reveal an extraordinary degree of divergence in variable domains
Source: BMC Evol Biol. 2008 Jun 19;8:177. doi: 10.1186/1471-2148-8-177 (PMC2442602; doi:10.1186/1471-2148-8-177)

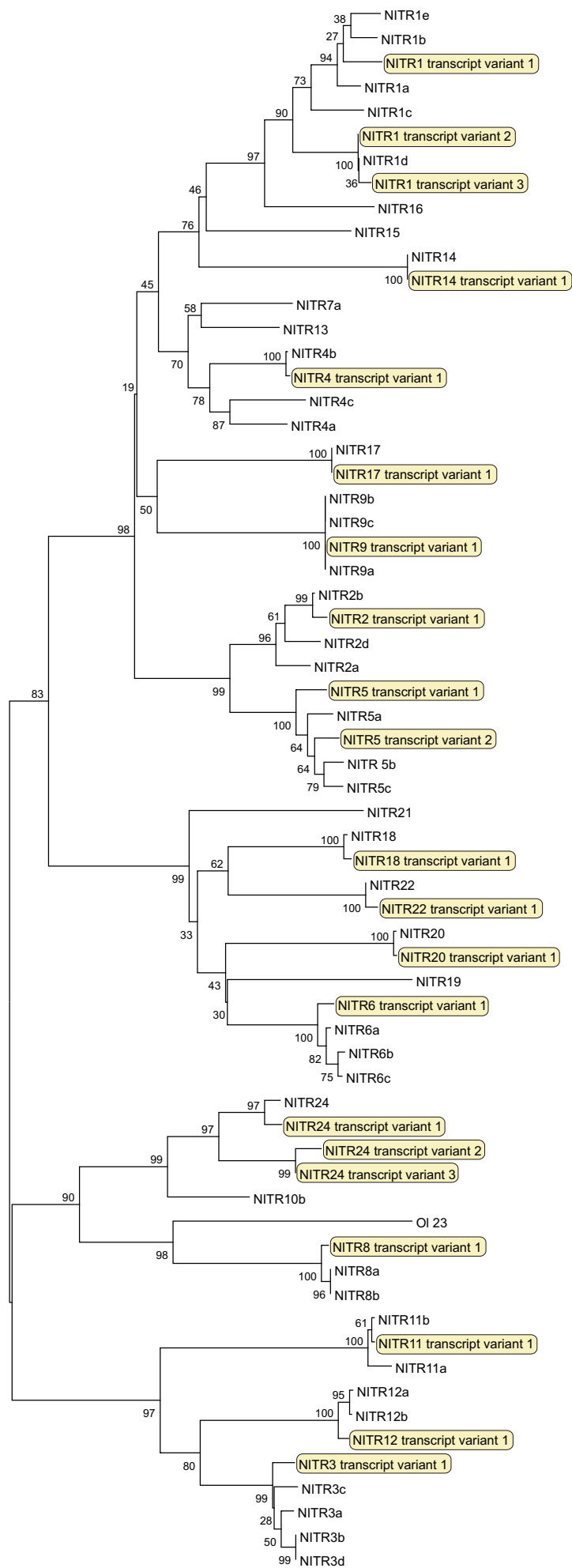

0.1

Supplement: Additional File 5 — Classification of medaka NITR cDNAs. Neighbor-joining tree of V domains encoded by out-bred, orange-red medaka NITR cDNAs (highlighted in yellow) and predicted NITR V domains from the genome of the inbred Hd-rR medaka. The number assigned to each interior branch corresponds to the bootstrap value. Branch lengths correspond to the number of amino acid substitutions estimated by Poisson correction, scale indicated below. [file 1471-2148-8-177-S5.pdf]

**a****Medaka Chr 5**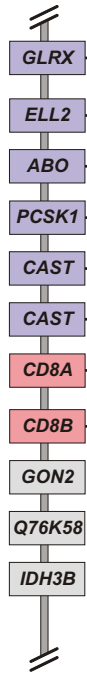**b****Zebrafish Chr 21**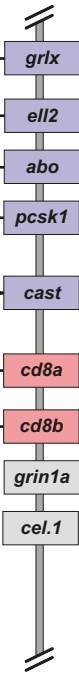**c****Stickleback Grp XVII**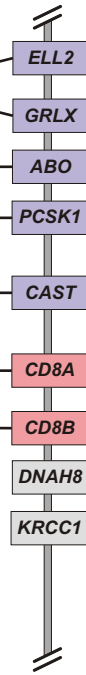**d****Human Chr 5**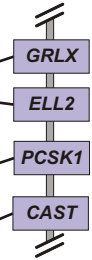**Human Chr 2**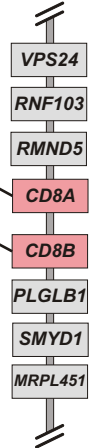

Supplement: Additional File 10 — The CD8 locus does not display conserved synteny between medaka, zebrafish, stickleback and human. Although the CD8A and CD8B genes are tightly linked in (a) medaka, (b) zebrafish, (c) stickleback and (d) human, flanking genes are not well conserved between fish and human. Data were acquired using release NCBI 36 of the human genome, release 1.0 of the medaka genome, release Zv7 of the zebrafish genome and release 1.0 of the stickleback genome. [file 1471-2148-8-177-S10.pdf]

**a**

**Human Chr 2q33**

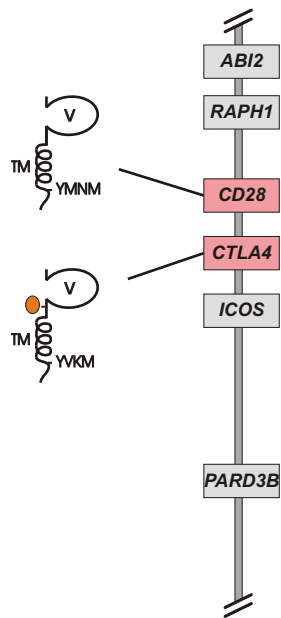

**b**

**Medaka Chr 21**

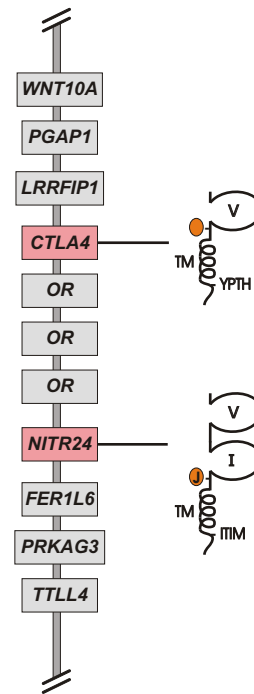

Supplement: Additional File 11 — Medaka NITR24 is linked to CTLA4. (a) Human CTLA4 and CD28 are tightly linked. (b) In medaka CTLA4 is closely linked to NITR24. Gene names and chromosomal order were identified via Contig View within the Ensembl Genome Browser [28]. Three predicted olfactory receptor (OR) genes are indicated on medaka chromosome 21, although the exact number of OR genes has not been determined. Data were acquired using release NCBI 36 of the human genome and release 1.0 of the medaka genome. CTLA4 and CD28 are not linked to NITR genes in zebrafish (not shown). Note that Ensembl misidentified medaka CTLA4 [18] as CD28. Predicted protein structures are shown and structural features are as in Figure 4 plus cytoplasmic activating motifs (YxxM); and a functionally unresolved motif (YxxH). [file 1471-2148-8-177-S11.pdf]

a

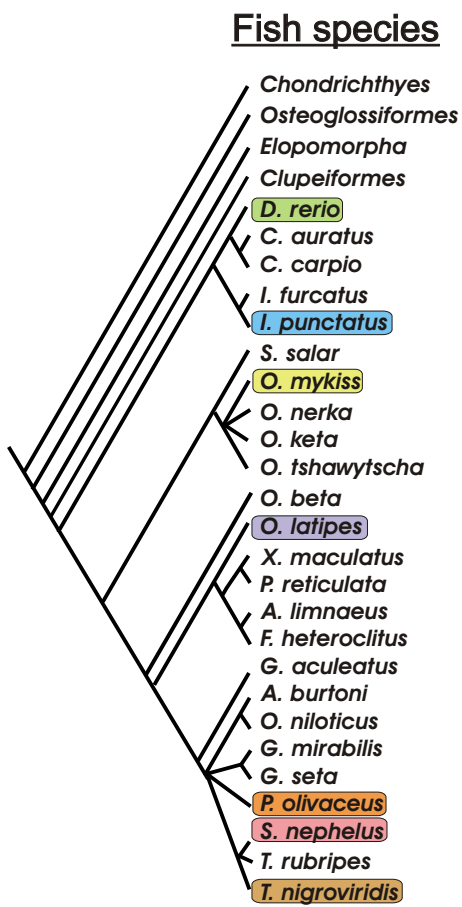

b

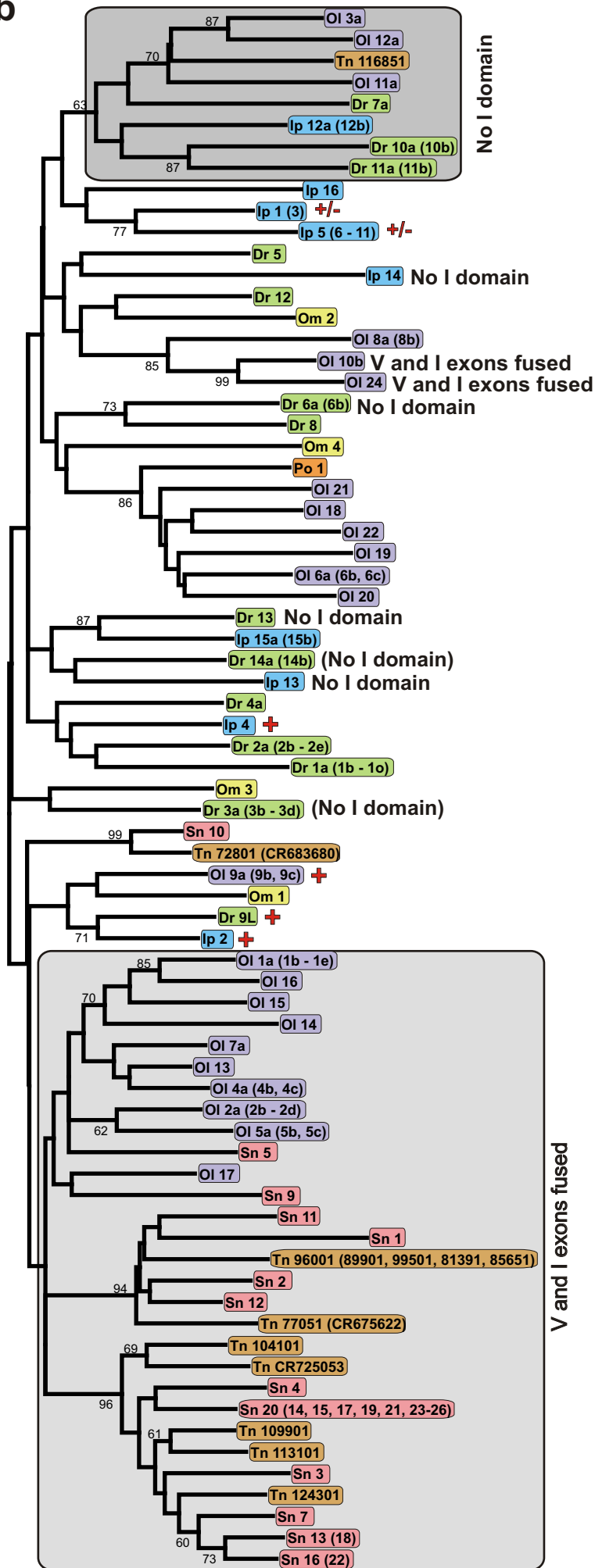

Supplement: Additional File 12 — Phylogenetic comparisons of published NITR V domains. (a) Cladogram depicts conventional phylogenetic radiations of bony fish [Figure adapted from 35]. Colors denote species in which NITR genes and/or gene transcripts have been characterized. (b) Neighbor-joining tree of V domains encoded by 73 representative NITR genes. Ol, medaka (Oryzias latipes); Dr, zebrafish (Danio rerio); Ip, channel catfish (Ictalurus punctatus); Om, rainbow trout (Oncorhynchus mykiss); Sn, Southern pufferfish (Spheroides nephelus); Po Japanese flounder (Paralichthys olivaceus) and Tn, pufferfish (Tetraodon nigroviridis). Specific NITR gene numbers are indicated (e.g. medaka NITR8a = Ol 8a), except for Tetraodon sequences (see Additional file 3). Only one NITR gene family member is incorporated; additional family members are indicated in parenthesis. Activating NITR genes are indicated by a "+"; NITR families that possess both inhibitory and activating forms are indicated by a "+/-". NITR genes which encode a V and I domain in a single exon ("V and I exons fused") and "No I domain" are indicated. These labels are presented in parentheses for NITR families, which do not consistently conform to these definitions. Number assigned to each interior branch corresponds to the bootstrap value; bootstrap values less than 60 are not shown. Branch lengths correspond to number of amino acid substitutions estimated by Poisson correction, indicated below. [file 1471-2148-8-177-S12.pdf]
