# Supplementary material for: The medaka novel immune-type receptor (NITR) gene clusters reveal an extraordinary degree of divergence in variable domains
Source: BMC Evol Biol. 2008 Jun 19;8:177. doi: 10.1186/1471-2148-8-177 (PMC2442602; doi:10.1186/1471-2148-8-177)
Supplement: Additional File 6 — Variation between the medaka NITR12 cDNA and genomic sequence. A partial alignment of NITR12 cDNA from out-bred, orange-red medaka to the predicted coding sequence of the NITR12a gene from inbred Hd-rR medaka. Predicted peptide sequence is shown above (cDNA) and below (genomic DNA) the alignment. The sequences shown include a portion of the transmembrane domain exon and the two cytoplasmic domain exons. Dashes indicate gaps in the nucleotide alignment. Asterisks indicate nucleotide identity. Stop codons are indicated by red highlighting in the nucleotide sequence and by dashes in the protein sequence. Downward arrowheads define predicted RNA splice sites. The NITR12 cDNA encodes additional nucleotide sequences that are not present in the genomic sequence (nucleotides highlighted in pink). Although the NITR12a genomic sequence is predicted to encode an ITIM (highlighted in yellow) and a second ITIM-like sequence (itim; highlighted in orange), differences in the NITR12 cDNA alter the predicted ITIM sequence (LNYAAL to RYYAAL) and introduce a stop codon, preventing translation of the itim. The differences in predicted protein sequence likely reflect allelic variations between the out-bred, orange-red transcripts and Hd-rR genomic sequence. [file 1471-2148-8-177-S6.pdf]

```
                    K  S  F  Q  K  A ▼ P  S  E  H  Q  H  T  E ▼ S  L  E  S  N  I
NITR12 cDNA        AAATCTTTTCAGAAGGCT CCGTCTGAGCACCAGCACACAGAG AGCCTGGAATCCAACATT
NITR12a genomic    AAATCTTTTCAGAAAGCT CCGTCTGAGCGCCAGCACAGAGAG AGCCTGGAATCCAACGTT
                   ************** *** ********** ******** **** *************** **
                    K  S  F  Q  K  A   P  S  E  R  Q  H  R  E   S  L  E  S  N  V

                    L  N  Y  A  A  L  T  F  Q  P  K  S  K  T  Q  R  G  P  G  P
NITR12 cDNA        CTTAACTATGCAGCCCTGACATTCCAGCCAAAATCTAAAACCCAGAGAGGTCCAGGACCT
NITR12a genomic    CGATACTATGCAGCCCTGACATTCCAGCCAAAACCTGAAACCCA----------------
                   *   ***************************** ** *******
                    R  Y  Y  A  A  L  T  F  Q  P  K  P  E  T  Q

                    E  T  -
NITR12 cDNA        GAGACTTAACCAGACCAAAGCATATCTGTATATAAAACAGATTCATCATCTGCTTTCTGA
NITR12a genomic    -----------------AAGCATATCTGTATATAAAACCGATTCA---------------
                                    ********************* ******
                                      S  I  S  V  Y  K  T  D  S

NITR12 cDNA        ACACTGGTGGGATTTCTGTTTCAAAATGATAAAAAGAGGAATTTGTCTGATGATTACATT
NITR12a genomic    ----------GATCTGTGTTT--------------------TTGGTCT------------
                             *** * *****                    ** ****
                              D  L  C  F                      W  S

NITR12 cDNA        CACCCTAGGTGTGAATGTAAGTGTGCATGGGTGTGTGATTGTGGCCCTGCGACAGACTGG
NITR12a genomic    -----TAG----------------------------------------------------
                        ***
                         -
```
