# Supplementary material for: The medaka novel immune-type receptor (NITR) gene clusters reveal an extraordinary degree of divergence in variable domains
Source: BMC Evol Biol. 2008 Jun 19;8:177. doi: 10.1186/1471-2148-8-177 (PMC2442602; doi:10.1186/1471-2148-8-177)
Supplement: Additional File 7 — Joining (J) and J-like sequences in medaka NITRs. The consensus joining (J) sequence is FGXGTXLX(V/L). A J-like sequence possesses either motif FGXG or TXLX(V/L/I) or partial sequence of both motifs. Sequences were compiled from version 1.0 of the Hd-rR genomic sequence. [file 1471-2148-8-177-S7.pdf]

J or J-like sequence	NITR V domain	NITR I domain
FGXGTXLXV		10b, 24
FGXGTXLXL		1a, 1b, 1c, 1d, 1e, 2a, 2b, 2d, 4a, 4b, 5a, 5b, 5c, 7a, 13, 14, 15
FGXGTXVXI		4c, 18, 22
FGXGTXVXL		17
FGXGTXVXM		20
FGXGTXLXM		9a
FGXGLXLXV	9a, 9b, 9c	
FGXXTXVXI		6a, 6b, 6c
FXXGTXLXV	8a, 8b	
FXXGVXVXV	7a	
FXXGSXLXV	6a, 6b, 6c	
GXGTXLXM		9b,9c
GXGTXLXE		8a, 8b
FGXG		21
TXLXV	3a, 3b, 3c, 3d, 12a, 12b, 23	
TXLXI	11a, 11b	
