# Supplementary material for: The medaka novel immune-type receptor (NITR) gene clusters reveal an extraordinary degree of divergence in variable domains
Source: BMC Evol Biol. 2008 Jun 19;8:177. doi: 10.1186/1471-2148-8-177 (PMC2442602; doi:10.1186/1471-2148-8-177)
Supplement: Additional File 8 — ITIM and ITIM-like sequences in medaka NITRs. Consensus ITIMs are defined as (S/I/V/L)xYxx(I/V/L) [33]. An ITIM-like sequence lacks a consensus residue in either the first or last position. Sequences were compiled from version 1.0 of the Hd-rR genomic sequence. [file 1471-2148-8-177-S8.pdf]

ITIM or ITIM-like sequence	Associated with V domain	Associated with I domain
LXYXXV	1a, 1b, 1c, 1d, 2b, 2d, 3a, 3b, 3c, 3d, 4a, 4b, 4c, 13, 14, 15	18
LXYXXL	5a, 5b, 5c, 7a, 11b, 19, 21	
LXYXXI	16	
IXYXXL	22	
SXYXXV	18	22
YXYXXV		1a
VXYXXA	12a, 12b	
LXYXXA	8a	
LXYXXP	8b	
CXYXXV		1b, 1c, 1d, 2b, 2d, 4a, 4b, 4c, 5c, 7a, 13, 14, 15, 16
AXYXXV		3a
VXYXXT	10b	3b, 3c, 11b
VXYXXR	11a	
TXYXXI		8b
SXYXXD		12a, 12b
MXYXXL	20	
