# Supplementary material for: The medaka novel immune-type receptor (NITR) gene clusters reveal an extraordinary degree of divergence in variable domains
Source: BMC Evol Biol. 2008 Jun 19;8:177. doi: 10.1186/1471-2148-8-177 (PMC2442602; doi:10.1186/1471-2148-8-177)
Supplement: Additional File 9 — Transmembrane domains of activating NITRs. Transmembrane domains predicted by SMART software [34]. Charged residues are white text on black. Zebrafish Nitr9 has been shown to partner with and signal via Dap12 [15]. Catfish IpNITR2, IpNITR3, IpNITR4, IpNITR10 and IpNITR11 encode transmembrane domains similar to medaka NITR9 and zebrafish Nitr9 [4]. [file 1471-2148-8-177-S9.pdf]

| Receptor | Transmembrane domain | Species shown |
|---|---|---|
| NITR9a, b, c | LSVIRTGVLLLFLLSCVLFV | Medaka |
| Nitr9 | LSIARTCLLLLVIVMVIIWYCVY | Zebrafish |
| IpNITR2 | FSIVRSVVLLCCVILIMIYFCCS | Channel catfish |
| IpNITR3 | LSVFRVGVLAFILIIFTMYYNVL | Channel catfish |
| IpNITR4 | LSIIRSAVLLLIFIICLLCTYIG | Channel catfish |
| IpNITR10/11 | LSIFRVGVLAFMLLIFPIILIVF | Channel catfish |
